# Digital CBT for insomnia and emotion regulation in the workplace: a randomised waitlist-controlled trial

**DOI:** 10.1017/S0033291725000194

**Published:** 2025-02-17

**Authors:** Talar R. Moukhtarian, Sophie Fletcher, Lukasz Walasek, Krishane Patel, Carla Toro, Anna L. Hurley-Wallace, Charlotte Kershaw, Sean Russel, Guy Daly, Nicole K. Y. Tang, Caroline Meyer

**Affiliations:** 1 Warwick Medical School, Division of Health Sciences, Mental Health and Wellbeing group, University of Warwick, Coventry, UK; 2 Department of Psychology, University of Warwick, Coventry, UK; 3 Natwest Group, London, UK; 4 Musculoskeletal Research Unit, Bristol Medical School, University of Bristol, Bristol, UK; 5 Faculty of Health and Life Sciences, Coventry University, Coventry, UK; 6 The British University in Egypt, Cairo, Egypt

**Keywords:** Insomnia, Cognitive Behavioural Therapy for Insomnia, Emotion Regulation, Randomised Controlled Trial, Workplace

## Abstract

**Background:**

Cognitive behavioural therapy for insomnia (CBT-I) is the recommended first-line treatment for insomnia. However, scaling this proven effective intervention to areas of high need remains a challenge, necessitating sensitive adaptation and evaluation.

**Methods:**

A randomised waitlist-controlled trial evaluated the efficacy of a hybrid digital CBT-I and emotion regulation (dCBT-I + ER) intervention delivered through workplaces. Participants with at least mild insomnia and depression or anxiety symptoms were randomised to the intervention or waitlist control groups. The intervention was delivered via a web-based platform and four video-conferencing therapy sessions. Participants tracked their sleep using actigraphy and a sleep diary that was used to pace the intervention delivered. Assessments occurred at baseline and 8 weeks post-randomisation, measuring insomnia, depression, anxiety, psychological well-being, quality of life, and work productivity.

**Results:**

Of the 159 participants (mean age 43.6 ± 9.4 years, 76.7% female, 80.5% white), 80 received the intervention and 79 were in the control group. The intervention group showed significant improvements in insomnia (F1, 134 = 71.46, p < .0001); depression (F1, 134 = 35.67, p < .0001); and anxiety (F1, 134 = 17.63, p < .0001), with large effect sizes (d = 0.7–1.5). Sleep diary data supported these findings, whereas actigraphy data did not. Improvements in psychological well-being were significant (F1, 132.13 = 10.64, p < 0.001), whereas quality of life, work productivity, and satisfaction outcomes were not.

**Conclusions:**

This study suggests that a hybrid dCBT-I + ER intervention, delivered via workplaces, effectively improves insomnia, depression, and anxiety. It holds promise as a scalable solution, warranting further investigation into its long-term efficacy and economic impact.

## Introduction

Insomnia, characterised by difficulties initiating or maintaining sleep causing daytime functional impairment (American Psychiatric Association, 2013), affects approximately 10% of the general population (Morphy, Dunn, Lewis, Boardman, & Croft, 2007), with 6% meeting diagnostic criteria for the disorder (Morin et al., 2011; Roth et al., 2011). This presents a significant public health concern, especially in the workplace (Rajaratnam, Licamele, & Birznieks, 2015; Yang et al., 2018), with links to increased accidents, absenteeism, presenteeism, sick leave, and reduced productivity (DiBonaventura et al., 2015; Hu, Hong, Yeh, & Hsieh, 2019; Laugsand, Strand, Vatten, Janszky, & Bjørngaard, 2014). These consequences of insomnia account for the loss of 200,000 working days annually, costing the UK economy approximately £50 billion each year, equivalent to 1.86% of the UK’s total GDP. Without intervention, this cost is projected to rise to around £60 billion (2.17% GDP) by 2030 (Hafner, Stepanek, Taylor, Troxel, & van Stolk, 2016).

Cognitive behavioural therapy for insomnia (CBT-I) is the recommended first-line treatment for insomnia according to international guidelines (Practitioner, 2015; Qaseem, Kansagara, Forciea, Cooke, & Denberg, 2016; Riemann et al., 2023). Meta-analyses indicate a moderate to large effect of CBT-I on insomnia and related sleep parameters (e.g. sleep efficiency [SE], sleep onset latency [SOL], wake after sleep onset [WASO], sleep quality) in people with and without comorbid psychiatric conditions (Riemann et al., 2023; Wu, Appleman, Salazar, & Ong, 2015).

In workplaces specifically, a recent meta-analysis of sleep interventions has shown moderate effects on reducing insomnia symptoms and some improvements in work productivity and presenteeism, but not absenteeism (Vega-Escaño et al., 2020). However, of the 12 studies included in the meta-analyses, only three were based on CBT. Therefore, although the literature reveals evidence of significant associations between insomnia and employees’ health and work outcomes, there are gaps in the research yet to be addressed.

Emotion regulation (ER) plays a vital role in connecting sleep quality with workplace outcomes. ER involves processes that influence our emotions, including when and how we express and experience them (Gross, 1998). Dysfunctions in the ER are common underlying factors in psychiatric disorders, such as depression and anxiety (Aldao, Nolen-Hoeksema, & Schweizer, 2010; American Psychiatric Association, 2013). Studies suggest that maladaptive ER contributes to insomnia, with rumination and worry maintaining insomnia symptoms (Gruber, Eidelman, & Harvey, 2008; Meneo, Samea, Tahmasian, & Baglioni, 2023; Van Someren, 2021). Longitudinal research indicates that increasing ER difficulties are associated with a higher risk of insomnia incidence or persistence over time (Jansson-Fröjmark, Norell-Clarke, & Linton, 2016).

Although CBT-I has demonstrated the efficacy in improving insomnia, it does not explicitly target ER. Interventions integrating ER strategies within CBT-I frameworks may offer enhanced benefits, addressing both the physiological and emotional mechanisms underlying insomnia. Emerging research indicates that ER is not merely an adjunctive consideration but may play a central role in optimising insomnia treatment (Riemann et al., 2023).

Interventions simultaneously addressing insomnia and ER are therefore needed. In this study, we adopt a hybrid approach of CBT-I targeting both ER and sleep problems with cognitive, behavioural and psychoeducation components, which is in line with the American Academy of Sleep Medicine clinical guidelines of treating insomnia with multicomponent CBT (Edinger et al., 2021). This integrated approach acknowledges that insomnia rarely occurs in isolation (Tang, Goodchild, & Salkovskis, 2012), and that targeting both insomnia and ER simultaneously, may improve treatment efficacy. Indeed, similar hybrid methods have shown promise in treating chronic pain and insomnia (Tang, 2018).

This study (SLEEP) evaluated the efficacy of a digital CBT-I + ER (dCBT-I + ER), designed to improve mild to severe insomnia and ER symptoms.

## Methods

### Research design

This study was a randomised controlled trial. Participants were assigned to CBT-I + ER or waitlist control by simple randomisation with a 1:1 allocation ratio. The computerised randomisation programme was devised and managed by a researcher (KP) who was blind to subsequent allocations. Participants were informed of their allocation outcome by the trial management team, who were the data controllers for personally identifiable data only (e.g. name, email). The rest of the research team and those involved in statistical analyses were blind to allocations and had access only to non-identifiable research data.

Screening, consent, questionnaire assessments, as well as delivery of intervention were all carried out online. The trial was registered (ISRCTN13596153) with the protocol published (Moukhtarian et al., 2022).

### Participants

Participants were recruited through organisations from the central part of the UK, the Midlands (part of a wider collaboration, the Mental Health and Productivity Pilot (MHPP) programme) or self-referred by social media advertising. Recruitment began on 12/4/21 and ended on 4/1/22. Participants were eligible if they were 18 or above, in employment, able to give informed consent, English speaking, and scored >7 on the Insomnia Severity Index (ISI), and >4 on either the General Anxiety Disorder-7 (GAD-7) or Patient Health Questionnaire-9 (PHQ-9). Refer to the published protocol (Moukhtarian et al., 2022) for the full list of eligibility criteria.

Nine hundred and two workers expressed interest in taking part in one of the MHPP trials (SLEEP was one of three trials advertised). Around 60% proceeded to complete the eligibility screen, and of those, 42% were eligible and invited to enrol in SLEEP. Then, 72% of those invited, consented to the trial and provided baseline measures with only 15% attrition until post-intervention (T2) across both groups (There was 9% drop out in the control arm and 20% in the treatment arm. Reasons cited for dropping out were; “unable to commit/no time”, “no longer needing help”, “aspect of trial undesirable”, “unwell and/or on holiday”, “left the workplace”, “treatment conflicting with other external support”, “no reason/did not contact research team”).

### Intervention

dCBTI+ER is a hybrid 8-week digital intervention programme. The self-guided component of the programme was hosted on a web-platform with weekly content expected to last around 1 hour. In addition, participants were offered four video-conferencing therapy sessions by trained therapists held over Microsoft Teams. For more information on the qualifications and training/supervision of therapists, see study protocol (Moukhtarian et al., 2022). The components of the intervention are behavioural (e.g. sleep restriction therapy, stimulus control therapy); cognitive (e.g. unhelpful thinking styles, cognitive reframing); educational (e.g. sleep science, sleep hygiene); and ER skills (e.g. relaxation, acceptance and commitment) in the form of interactive psychoeducation, skills training, exercises, and homework.

### Measurements

Quantitative outcomes were assessed using self-report questionnaires and administered at weeks 0 (baseline) and 8 (end of treatment), with short- and long-term follow-ups at months 1, 6, and 12 (Follow-up analyses are not reported in this publication). On Qualtrics. Sleep parameters (self-reported sleep diary and actigraphy data) were collected over the same 1 week at baseline and end of treatment.

The primary outcomes were the symptoms of insomnia, anxiety, and depression measured, respectively, by the ISI (Bastien, Vallières, & Morin, 2001), GAD-7 (Spitzer, Kroenke, Williams, & Löwe, 2006), and PHQ-9 (Spitzer, Kroenke, & Williams, 1999).

The secondary outcomes were the following standardised measures with more details on each in the published protocol: (1) Work productivity, measured through the Work Productivity and Activity Impairment: General Health v2.0 (WPAI:GH) (Reilly, Zbrozek, & Dukes, 1993); (2) Job satisfaction, measured using the Indiana Job Satisfaction Scale (Resnick & Bond, 2001); (3) Well-being – measured using the Warwick-Edinburgh Mental Health Well-being Scale (WEMWBS) (Tennant et al., 2007); (4) Quality of life- measured using the EuroQOL EQ-5D-5L questionnaire (Herdman et al., 2011)^1^.

Self-reported data on sleep quality, bedtime, waking and out-of-bed times, SOL, and total WASO were collected using a modified version of the validated Consensus Sleep Diary (Carney et al., 2012). The total sleep time (TST), total time in bed (TIB), and SE were calculated based on these parameters. Actigraphy monitoring was conducted using the MotionWatch8 device supplied by CamNtech. Data from these devices were cleaned and processed using the MotionWare software. We implemented strategies to minimise artefacts in actigraphy data, including data cleaning, manual review of unclear segments, and exclusion of flagged data when necessary. Participants were instructed to wear the device continuously and document the removal periods, which were cross-referenced with actigraphy data for verification and adjustment. Participants were also excluded from these analyses if they had fewer than three nights of sleep data at each timepoint. Actigraphy outcomes included TIB, SE, wake bouts, sleep bouts, total wake time (TWT; calculated as the sum of WASO and SOL), and TST. It is important to note that SOL was not reported in this study as a standalone variable due to the known limitations of actigraphy in accurately estimating this parameter (Walia & Mehra, 2019). Actigraphy is well-validated for deriving metrics such as TST, SE, and WASO; however, its reliability for measuring SOL is less consistent when compared to polysomnography. As noted in previous studies, including Ancoli-Israel et al. (2003), the correlation between actigraphy and PSG for SOL estimation demonstrates weaker reliability, with coefficients ranging from r = 0.89 to 0.98. Given these limitations, we prioritised reporting metrics with stronger empirical validation to ensure the accuracy and reliability of our findings.

User engagement was examined for each participant for their overall usage (percentage of completion) of the online platform (over the 6-weeks period) and attendance of therapy appointments (of a total four offered).

A qualitative process evaluation looking at the barriers and facilitators of engagement was conducted and is reported in a separate publication (Hurley-Wallace et al., 2022).

No serious adverse event was reported, and adverse events reported were all assessed to be unrelated to the intervention. A table summarising the events reported during the trial are in Supplementary Material, Appendix 1.

### Statistical analysis

Sample size was determined based on a previous hybrid CBT study in chronic pain patients (Tang et al., 2020), which reported a large effect size (Cohen’s *d* = 1.73; Hedges’ *g* = 1.73) for hybrid CBT versus self-help control on ISI at 12-weeks follow-up. However, given the small sample and a more heterogeneous non-clinical sample (symptom severity and individual characteristics), a more conservative effect size of *d* = 0.5 in ISI was used. Using a standard significance level (*p* = 0.05) with default statistical power (0.8) and a small interclass correlation (ICC = .03), we calculated the need for 130 participants, with 65 in each of the CBT-I + ER and control groups with a 1:1 allocation ratio. To counteract attrition (20%), the sample size was increased to 156 (76 in each arm) to ensure adequate statistical power.

Given the three primary outcome measures (ISI, PHQ-9, GAD-7), we run models adjusting for an inflated error rate by using a Bonferroni corrected significance threshold (α = 0.05/3 = 0.01667). The analyses were conducted using R (Team, 2022) and SPSS version 29 (Inc, 1998).

An outline of the analysis strategy is provided in the published trial protocol (Moukhtarian et al., 2022), with additional steps described below.

Having checked that assumptions of normality were met (see Supplementary Material, Appendix 2), group differences in baseline demographics, primary, and secondary outcomes were examined using *t* tests for continuous variables and Chi-square tests for categorical variables, or their non-parametric equivalents for non-normally distributed data.

Primary outcomes were analysed using linear mixed effects models, incorporating group allocation (treatment and waitlist control) and assessment timepoint (baseline and end of treatment) as fixed effects, along with an interaction term between timepoint and allocation in each model. The model included participant-level effect as random intercept. Results are presented with 95% CIs and two-sided *p* values, as well as Cohen’s *d* standardised effect sizes (McGough & Faraone, 2009). Secondary outcomes were analysed using equivalent linear mixed effects models.

Analyses initially focused on complete cases, including participants with recorded outcomes at baseline and 8 weeks^2^. Missing data were imputed using the MICE technique (assuming missing at random (MAR), supported by analyses in Supplementary Material, Appendix 3). Primary outcome models were then repeated using an intention to treat (ITT) approach, including all randomised participants. The robustness of assumptions regarding missing outcome data was assessed by multinomial logistic regression models using missingness as dependent variable, allocation as a factor and each of the outcome measures as covariates to predict missingness with the variables at baseline (i.e. does symptom severity on ISI at baseline predict missingness/drop out at week 8). Results showed that the overall attrition at post-intervention was only 15% and none of the variables predicted missingness except for three subscales of the WPAI:GH. MAR assumption was therefore upheld. Full results reported in Supplementary Material, Appendix 3.

Further, treatment efficacy on the primary outcomes was examined by analysing changes from caseness to non-caseness (i.e. from above to below clinical cut-offs) at baseline and after intervention/waitlist, as well as rates of clinically significant change (CSC) and recovery. Details on caseness and CSC definitions are provided in Supplementary Material, Appendix 4.

### Ethical approval and consent

The authors assert that all procedures contributing to this work comply with the ethical standards of the relevant national and institutional committees on human experimentation and with the Helsinki Declaration of 1975, as revised in 2008. All procedures involving human subjects/patients were approved by the University of Warwick Biomedical and Research Ethics Committee (BSREC 45/20-21 AM04).

## Results

**Figure 1.**
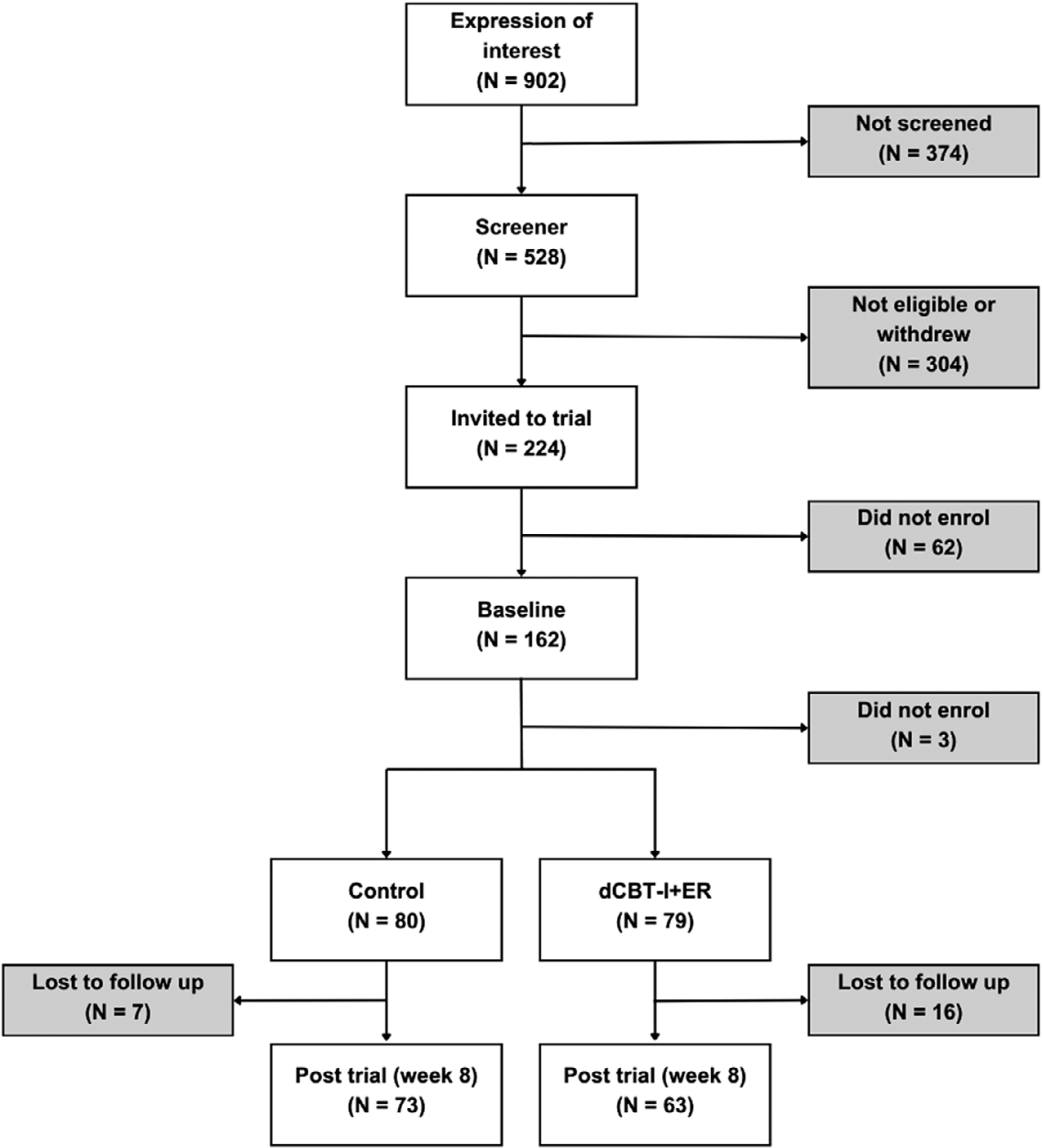
Recruitment flow chart.

Following the EQUATOR Reporting Guidelines, we recorded and reported all participants flow. Figure 1 is a flow chart that shows the numbers of participants at each stage in the process.

The final sample included 159 adults, of whom 122 (76.7%) were female, 128 (80.5%) were white, and the mean (SD) age was 43.6 (9.4) years.

Summary descriptive statistics and group differences at baseline for demographic, primary, and secondary outcomes are presented in Tables 1 and 2. There were no significant differences between groups at baseline on demographic, primary, or secondary variables.

**Table 1.** Baseline characteristics

	dCBT-I + ER (n = 79)	Control (n = 80)	Test statistics
**Demographics**			
Age, y	44.59 (9.11)	42.55 (9.67)	*t*(157) = −1.37, *p* = 0.172
Sex, No. (%)			*X^2^* = 0.37, *p* = 0.544
Women	59 (74.70)	63 (78.80)	
Men, Other	20 (25.30)	17 (21.30)	
Ethnicity, No. (%)			*X^2^* = 0.03, *p* = 0.872
White	64 (81.00)	64 (80.00)	
Black, Asian, Mixed ethnicity, Other	15 (19.00)	16 (20.00)	
Relationship status, No. (%)			*X^2^* = 1.64, *p* = 0.651
Married	39 (49.40)	43 (53.80)	
Cohabiting	14 (17.70)	16 (20.00)	
Single	14 (17.70)	14 (17.50)	
Divorced, separated, widowed, other	12 (15.20)	7 (8.80)	
Hours of work	37.58 (6.68)	37.31 (6.67)	*t*(157) = −0.26, *p* = 0.799
Education level, No. (%)			*X^2^* = 2.33, *p* = 0.127
Higher education^c^	49 (62.00)	40 (50.00)	
Lower education^d^	30 (38.00))	40 (50.00)	
Income No. (%)			*X^2^* = 2.38, *p* = 0.123
£10,000–£29,999	12 (15.20)	20 (25.00)	
£30,000 or higher	67 (84.80)	60 (75.00)	
**Primary outcomes**			
Insomnia (ISI)	16.34 (4.13)	15.65 (4.38)	*t*(157) = −1.03, *p* = 0.307
Depression (PHQ–9)	9.91 (4.47)	10.39 (4.63)	*U =* 3055.50, *p* = 0.718
Anxiety (GAD–7)	8.70 (4.56)	9.13 (4.31)	*U =* 2875.50, *p* = 0.325
*Categorical primary outcomes*			
Insomnia (ISI)			X^2^ = 2.66, *p* = 0.264
No clinically significant insomnia	2 (2.50)	4 (5.00)	
Subthreshold insomnia	23 (29.10)	31 (38.80)	
Clinical insomnia	54 (68.40)	45 (56.30)	
Depression (PHQ–9)			*X^2^* = 3.39, *p* = 0.494
Normal	9 (11.40)	4 (5.00)	
Mild	31 (39.20)	36 (45.00)	
Mild–moderate	26 (32.90)	28 (35.00)	
Moderate	10 (12.70)	7 (8.80)	
Severe	3 (3.80)	5 (6.30)	
Anxiety (GAD–7)			*X^2^* = 3.01, *p* = 0.390
No to low risk	15 (19.00)	10 (12.50)	
Mild	35 (44.30)	34 (42.50)	
Moderate	18 (22.80)	27 (33.80)	
Severe	11 (13.90)	9 (11.30)	
**Secondary outcomes**			
Work productivity (WPAI:GH)			
WPAIGH-WTM^a^	2.08 (4.13)	6.04 (19.52)	*U =* 2887.00, *p* = 0.178
WPAIGH-IWW	34.18 (26.87)	38.25 (27.41)	*U =* 2901.50, *p* = 0.369
WPAIGH-OWI^a^	34.11 (26.86)	39.73 (28.63)	*U =* 2781.50, *p* = 0.236
WPAIGH-AI	36.84 (27.15)	42.13 (29.33)	*U =* 2825.00, *p* = 0.245
Job satisfaction (IJSS)	3.01 (0.39)	2.98 (0.38)	*t*(157) = −0.473, *p* = 0.637
Well-being (WEMWBS)^b^	41.92 (7.29)	40.79 (7.66)	*t*(154) = −0.942, *p* = 0.348
Quality of life (EQ–5D–5L)			
EQ–5D–5L utility score^e^	0.82 (0.12)	0.79 (0.15)	*U =* 3254.00, *p* = 0.363
EQ–5D–5L VAS^f^	69.91 (17.81)	67.11 (18.80)	*U =* 3251.50, *p* = 0.452
**Actigraphy**			
*N*	52	67	
Total time in bed (hh:mm)	08:20 (00:64)	08:09 (00:55)	*U =* 1868.50, *p* = 0.498
Total wake time (min)	69.08 (34.08)	67.45 (34.92)	*U =* 1814.50, *p* = 0.698
Total sleep time (hh:mm)	06:51 (47.73)	06:48 (00:51)	*t*(117) = −0.342, *p* = 0.733
Sleep efficiency (%)	83.05 (6.93)	83.02 (6.97)	*U =* 1724.50, *p* = 0.925
**Sleep diary**			
N	47	56	
Total time in bed (hh:mm)	08:15 (01:04)	08:33 (00:56)	*U =* 1171.00, *p* = 0.337
Sleep onset latency (min)	25.01 (18.96)	27.73 (18.63)	*U =* 1164.00, *p* = 0.314
Wake after sleep onset (min)	42.70 (32.04)	43.26 (35.25)	*U =* 1344.00, *p* = 0.853
Total sleep time (hh:mm)	06:29 (01:04)	06:49 (01:11)	*t*(101) = 1.495, *p* = 0.138
Sleep efficiency (%)	79.03 (9.52)	79.55 (10.63)	*U =* 1240.00, *p* = 0.615
Sleep quality^g^	2.92 (0.51)	2.94 (0.59)	*t*(100) = 0.178, *p* = 0.430

a dCBT-I + ER (n = 78); Control (n = 80).

b dCBT-I + ER (n = 78); Control (n = 78).

c Higher education = Bachelor’s degree, Master’s degree, Doctorate degree.

d Lower education = Secondary school qualification, some diploma, other qualification, no qualification.

e dCBT-I + ER (n = 75); Control (n = 80).

f dCBT-I + ER (n = 76); Control (n = 80).

g dCBT-I + ER (n = 46); Control (n = 56)

*Notes*: Mean values are presented with standard deviations in parentheses unless otherwise specified. The test statistics results are from *t* tests for continuous variables, and Pearson *χ*^2^ tests for categorical variables.

*Abbreviatiions*: ISI, Insomnia Severity Index; PHQ-9, Patient Health Questionnaire; GAD-7, Generalised Anxiety Disorder scale; WPAI:GH, Work Productivity and Activity Impairment: General Health; WTM, Work Time Missed, IWW, Impairment While Working; OWI, Overall Work Impairment; AI, Activity Impairment; IJSS, Indiana Job Satisfaction Scale; WEMWBS, Warwick-Edinburgh Mental Health Well-being Scale; EQ-5D-5L VAS, EuroQOL EQ-5D-5L Visual Analogue Scale.

**Table 2.** Effects of dCBT-I + ER versus waitlist control on primary outcomes: Insomnia, depression and anxiety symptoms

	EMM (SE)				
	dCBT-I + ER	Waitlist control	Adjusted difference (95% CI)	*p* value	Cohen’s *d*
***Case complete***					
**ISI**					
Baseline	16.38 (0.58)	15.40 (0.54)	−0.98 (−3.03; 1.06)	0.5981	−0.304
Week 8	8.38 (0.58)	14.05 (0.54)	5.67 (3.63; 7.72)	**<.0001**	1.752
**PHQ–9**					
Baseline	10.02 (0.57)	10.36 (0.53)	0.34 (−1.68; 2.36)	0.9719	0.124
Week 8	5 (0.57)	9.33 (0.53)	4.33 (2.31; 6.34)	**<.0001**	1.576
**GAD–7**					
Baseline	8.84 (0.56)	9.23 (0.52)	0.39 (−1.61; 2.39)	0.9570	0.148
Week 8	5.29 (0.56)	8.38 (0.52)	3.10 (1.10; 5.09)	**0.0005**	1.169
***ITT***					
**ISI**					
Baseline	16.34 (0.46)	15.65 (0.46)	−0.692 (−2.378; 0.994)	0.7115	−0.268
Week 8	8.95 (0.46)	14.12 (0.46)	5.171 (3.485; 6.856)	**<.0001**	2.006
**PHQ–9**					
Baseline	9.91 (0.47)	10.39 (0.47)	0.476 (−1.255; 2.21)	0.8915	0.224
Week 8	5.44 (0.47)	9.24 (0.47)	3.802 (2.071; 5.53)	**<.0001**	1.790
**GAD–7**					
Baseline	8.70 (0.46)	9.12 (0.45)	0.429 (−1.248; 2.11)	0.9105	0.217
Week 8	5.46 (0.46)	8.20 (0.45)	2.739 (1.063; 4.42)	**0.0002**	1.388

*Abbreviations*: dCBT-I + ER, digital Cognitive Behavioural Therapy for Insomnia and Emotion Regulation; ISI, Insomnia Severity Index; PHQ-9, Patient Health Questionnaire; GAD-7, Generalised Anxiety Disorder scale.

### Treatment adherence

Participants in the intervention arm had good adherence to treatment with 73% overall platform content completion rate and a mean of 3.36 therapy appointments attended of a total of four.

## Efficacy of dCBT-I + ER versus waitlist control on primary outcomes

Table 2 presents the estimated marginal means (EMM) and standard errors (SE) of primary outcomes at baseline and week 8 for intervention and control groups, along with adjusted differences, *p* value and effect sizes (Cohen’s *d*) using case complete approach followed by ITT.

Significant *group* × *time* interactions were observed for ISI (F(1, 134) = 71.46, *p* < .0001); PHQ-9 (F(1, 134) = 35.67, *p* < .0001); and GAD-7 (F(1, 134) = 17.63, *p* < .0001), indicating significantly higher pre to post treatment symptom reductions in the dCBT-I + ER group compared to controls, all reaching Bonferroni corrected significance at *p* = 0.01667. Effect sizes were large for insomnia (d = 1.752), depression (d = 1.576), and anxiety (d = 1.169) severity reduction in the intervention group.

ITT sensitivity analyses on primary outcomes using an ITT approach. Significant group × time interactions were observed for ISI (F(1, 1429) = 513.79, *p* < .001); PHQ-9 (F(1, 1429) = 243.53, *p* < .001); and GAD-7 (F(1, 1429) = 136.20, *p* < .001), indicating greater symptom reductions in the dCBT-I + ER group compared to controls at week 8.

Results of the ITT models aligned with case complete analyses, showing similar effect sizes across all three outcomes.

## Case complete analyses on secondary outcomes

Table 3 displays the model outputs for secondary outcomes. Only the WEMWBS well-being scale showed improvements the dCBT-I + ER group (F(1, 132.13) = 10.64, *p* = 0.0014, *d* = 1.05). The non-significant interactions are reported in Supplementary Material, Appendix 5.

**Table 3.** Effects of dCBT-I + ER versus waitlist control on questionnaire-based secondary outcomes and sleep parameters

	EMM (SE)				
	dCBT-I + ER	Waitlist control	Adjusted difference (95% CI)	*p* value	Cohen’s *d*
**WPAI-WTM**					
Baseline	1.23 (1.53)	3.29 (1.41)	2.06 (−3.32; 7.44)	0.7559	0.173
Week 8	3.76 (1.54)	3.44 (1.42)	−0.32 (−5.74; 5.11)	0.9988	−0.027
**WPAI-IWW**					
Baseline	32.90 (3.27)	36.00 (3.03)	3.17 (−8.37; 14.70)	0.8926	0.603
Week 8	21.30 (3.27)	31.20 (3.03)	9.96 (−1.57; 21.50)	0.1170	0.948
**WPAI-OWI**					
Baseline	32.50 (3.41)	37.50 (3.14)	4.94 (−7.07; 16.90)	0.7117	0.240
Week 8	22.70 (3.44)	33.00 (3.16)	10.22 (−1.87; 22.30)	0.1297	0.496
**WPAI-AI**					
Baseline	37.30 (3.52)	40.70 (3.27)	3.38 (−9.05; 15.80)	0.8951	0.662
Week 8	23.70 (3.52)	37.40 (3.27)	13.75 (1.32; 26.20)	0.0237	1.200
**IJSS**					
Baseline	3.02 (0.05)	2.97 (0.05)	−0.05 (−0.24; 0.13)	0.8684	−0.291
Week 8	3.02 (0.05)	2.95 (0.05)	−0.07 (−0.26; 0.11)	0.7342	−0.388
**WEMWBS**					
Baseline	41.70 (0.99)	40.70 (0.92)	−1.06 (−4.56; 2.44)	0.8609	−0.250
Week 8	46.40 (0.99)	41.90 (0.92)	−4.45 (−7.94; −0.96)	**0.0062**	−1.048
**EQ–5D–5L-Utility score**					
Baseline	0.81 (0.02)	0.80 (0.02)	−0.02 (−0.07; 0.04)	0.878	−0.226
Week 8	0.84 (0.02)	0.80 (0.02)	−0.04 (−0.10; 0.02)	0.248	−0.561
**EQ5D–5L-VAS**					
Baseline	68.90 (2.27)	67.90 (2.08)	−1.06 (−9.04; 6.92)	0.9860	−0.100
Week 8	75.90 (2.24)	70.10 (2.09)	−5.84 (−13.78; 2.09)	0.2278	−0.551
*Actigraphy*					
**Time in bed (hh:mm)**					
Baseline	08:23 (00:08)	08:10 (00:07)	−12.46 (−40.31; 15.40)	0.6528	−0.335
Week 8	07:48 (00:08)	08:18 (00:07)	30.23 (2.37; 58.09)	0.0277	0.812
**Total wake time (min)**					
Baseline	69.60 (4.49)	67.50 (4.00)	−2.08 (−17.71; 13.55)	0.9857	−0.129
Week 8	58.00 (4.49)	68.70 (4.00)	10.62 (−5.01; 26.25)	0.2942	0.658
**Total sleep time (hh:mm)**					
Baseline	06:53 (00:07)	06:49 (00:06)	−3.95 (−27.79; 19.90)	0.9732	−0.121
Week 8	06:30 (00:07)	06:50 (00:06)	19.75 (−4.09; 43.60)	0.1418	0.605
**Sleep efficiency (%)**					
Baseline	83.00 (0.95)	83.00 (0.85)	0.05 (−3.25; 3.36)	1.0000	0.014
Week 8	83.80 (0.95)	82.70 (0.85)	−1.11 (−4.41; 2.20)	0.8204	−0.297
*Sleep diary*					
**Time in bed (hh:mm)**					
Baseline	08:15 (00:08)	08:33 (00:08)	17.70 (−11.30; 46.70)	0.3872	0.507
Week 8	07:37 (00:08)	08:30 (00:08)	53.50 (24.50; 82.50)	**<.0001**	1.53
**Sleep onset latency (min)**					
Baseline	25.00 (2.80)	27.70 (2.57)	2.72 (−7.15; 12.59)	0.8912	0.202
Week 8	13.80 (2.80)	29.10 (2.57)	15.33 (5.46; 25.20)	**0.0005**	1.138
**Wake after sleep onset (min)**					
Baseline	42.70 (4.37)	43.30 (4.01)	0.55 (−14.83; 15.90)	0.9997	0.023
Week 8	20.00 (4.37)	38.00 (4.01)	17.99 (2.61; 33.40)	**0.0147**	0.751
**Total sleep time (hh:mm)**					
Baseline	06:29 (00:10)	06:49 (00:09)	20.18 (−13.60; 54.00)	0.4102	0.4816
Week 8	06:40 (00:10)	06:51 (00:09)	10.64 (−23.20; 44.50)	0.8463	0.2538
**Sleep efficiency (%)**					
Baseline	79.00 (1.46)	79.50 (1.33)	0.52 (−4.60; 5.65)	0.9935	0.066
Week 8	87.80 (1.46)	80.50 (1.33)	−7.31 (−12.43; 2.18)	**0.0016**	−0.924
**Sleep quality**					
Baseline	2.92 (0.09)	2.94 (0.08)	0.03 (−0.29; 0.35)	0.9968	0.055
Week 8	3.35 (0.09)	2.88 (0.08)	−0.47 (−0.79; 0.15)	**0.0010**	−1.009

*Abbreviations*: dCBT-I + ER, digital Cognitive Behavioural Therapy for Insomnia and Emotion Regulation; WPAI:GH, Work Productivity and Activity Impairment: General Health; WTM, Work Time Missed, IWW, Impairment While Working; OWI, Overall Work Impairment; AI, Activity Impairment; IJSS, Indiana Job Satisfaction Scale; WEMWBS, Warwick-Edinburgh Mental Health Well-being Scale; EQ-5D-5L VAS, EuroQOL EQ-5D-5L Visual Analogue Scale.

For actigraphy data, *Group* × *time* interactions for time in bed (F(1, 113) = 18.65, *p* < 0.0001); TWT (F(1, 113) = 8.79, *p* = 0.0037); and TST (F(1, 113) = 7.48, *p =* 0.0072), but not for SE (F(1, 113) = 1.37, *p* = 0.2441), However, no significant differences were observed between dCBT-I + ER and the control group on actigraphy variables at 8 weeks.

Similarly, all sleep diary data interaction models reached significance except for TST (F(1, 101) = 0.66, *p =* 0.4175). Significant interactions of other parameters are reported in Supplementary Material, Appendix 5. Furthermore, significant improvement on all sleep diary variables, except for TST, was observed in the dCBT-I + ER group compared to the control group at 8 weeks, with large effect sizes ranging from 0.7 to 1.5.

## Caseness, CSC, and recovery

Table 4 displays caseness frequencies by allocation at baseline (T1) and week 8 (T2) for primary outcomes. In the dCBT-I + ER group, there was a 45% remission in insomnia caseness, compared with 3% in the control group. Remissions in depression and anxiety caseness were approximately 30% in the dCBT-I + ER group, versus 3% and 10%, respectively, in the control group.

**Table 4.** Caseness at baseline and T2 for primary outcomes, and CSC from baseline to T2 across the groups^a^

	Caseness				CSC		
	Baseline (T1)		End-of-treatment (T2)		n (%)		
	dCBT-I + ER (n = 79)	Waitlist control (n = 80)	dCBT-I + ER (n = 63)	Waitlist control (n = 73)	dCBT-I + ER	Waitlist control	Test statistic
**ISI**	77 (97.50)	76 (95.00)	32 (50.80)	67 (91.80)	36 (57.10)	5 (6.80)	X^2^ = 40.62, ***p* < .001**
**PHQ–9**	33 (41.80)	35 (43.80)	8 (12.70)	30 (41.10)	28 (44.40)	5 (6.80)	X^2^ = 26.01, ***p* < .001**
**GAD–7**	34 (43.00)	41 (51.20)	9 (14.3)	30 (41.10)	26 (41.30)	14 (19.20)	X^2^ = 7.95, ***p =* .005**

a CSC scores were calculated only if T2 data were available. Analysis based on case complete dataset (no data were imputed).

*Abbreviations*: ISI, Insomnia Severity Index; PHQ-9, Patient Health Questionnaire; GAD-7, Generalised Anxiety Disorder scale; dCBT-I + ER, digital Cognitive Behavioural Therapy for Insomnia and Emotion Regulation.

The table also presents the rates of CSC in both groups. The dCBT-I + ER group showed significantly higher rates of treatment response compared to controls, with 57.10% versus 6.80% for ISI, 44% versus 6.80% for PHQ-9, and 41.30% versus 19.20% for GAD-7.

## Discussion

### Main findings

This study evaluated the efficacy of a hybrid dCBT-I + ER programme offered to employees in workplaces on insomnia, depression, anxiety, other well-being outcomes, and productivity. At baseline, participants exhibited moderately severe insomnia, mild to moderate depression, and anxiety. The intervention significantly improved all primary outcomes in the dCBT-I + ER group, with large effect sizes; *d* = 1.7 for insomnia, *d* = 1.6 for depression, and *d* = 1.2 for anxiety.

Productivity outcomes did not show significance; however, these outcomes were assessed as secondary with the intervention primarily focusing on insomnia and mental health, with assessments conducted immediately post-treatment. Improvements in sleep and ER may require more time to translate to improvements in productivity. Larger trials with longer follow-ups could explore this further.

This trial introduces a hybrid programme that merges CBT for insomnia with ER techniques in workplace settings. Results contribute to the existing literature supporting the efficacy of dCBT-I in reducing insomnia symptoms (Soh, Ho, Ho, & Tam, 2020). The intervention also significantly improved depression and anxiety symptoms, which were likely attributable to both the CBT-I and ER components. Notably, improvements in depression (Richards & Richardson, 2012) and anxiety (Romijn et al., 2019) exceeded those typically observed in digital interventions targeting these specific conditions, which may highlight the foundational role of sleep in mental health. Indeed, poor sleep is well-established as a bidirectional risk factor for both depression and anxiety, with chronic sleep disturbances increasing vulnerability to these conditions through mechanisms such as dysregulated emotional processing, heightened stress reactivity, and altered neurochemical balance. By addressing insomnia, the intervention may have tackled a core underlying driver of these mental health issues. These findings align with previous randomised controlled trials (Freeman et al., 2017) and systematic reviews (Ye et al., 2015) that have demonstrated significant and large improvements in depression and anxiety symptoms in CBT-I trials primarily aimed at treating insomnia. The results underscore the value of sleep interventions not only as effective standalone treatments but also as powerful adjuncts in mental healthcare, addressing the overlapping mechanisms that contribute to sleep and mental health disorders.

Finally, additional analyses assessing treatment response and remission also showed significant reductions within the dCBT-I + ER group; a 50% decrease in clinical insomnia and a 30% decrease in depression and anxiety cases post-intervention compared with baseline.

### Strengths and limitations

This dCBT-I + ER programme emerges as an effective solution to address the high prevalence of insomnia and its impact on the workforce, offering scalability and accessibility beyond traditional methods (Espie & Henry, 2023). Its remote format may have mitigated the stigma associated with seeking psychological support publicly. Unlike standard NHS Talking Therapies case management, targeting multiple symptom domains simultaneously reduced insomnia, depression, and anxiety. The drop-out rates at 8 weeks were approximately 15%; far less than the reported rates of other digital intentions of up to 40% (Ho et al., 2015), and ITT analyses confirmed the robustness of the primary models’ despite missing data. The excellent retention rates in this trial could be attributed similarly to the timing of the trial during the COVID-19 pandemic and its fully online format. During this period, employees were able to work from home with greater flexibility, which may have increased participants’ availability and willingness to engage in digital interventions due to reduced external commitments and a greater focus on health and well-being while at home. Additionally, the trial incorporated four therapy appointments during the 8-week study period, with appointment reminders sent in advance, which may have provided a sense of accountability and personalised support, enhancing engagement. These touch points likely mitigated the sense of isolation often associated with digital interventions and reinforced participants’ commitment to the programme, as evidenced by the low drop-out rates; unlike higher rates seen in fully self-guided digital interventions.

However, several limitations should be considered. First, the intervention platform could only show the overall content completion rate at week 8, with limited comprehensive user engagement data. Second, the sample skewed heavily towards females and white respondents. Third, the study used a waitlist (passive) control group. Finally, the study occurred during the COVID-19 pandemic, potentially affecting the results, warranting replication and longer-term follow-up.

Objective and self-reported sleep parameters yielded incongruent results, highlighting a discrepancy commonly observed between different assessment methods. It is often reported that these different assessment methods are only scarcely correlated with one another (Fernandez-Mendoza et al., 2011; Jackowska, Ronaldson, Brown, & Steptoe, 2016). This discrepancy may be related to the different assessment methods tapping into different dimensions of the sleep experience (Harvey & Tang, 2012).For example, self-reported inability to fall asleep or stay asleep does not often demonstrate objective sleep problems (Fernandez-Mendoza et al., 2011; Kay, Buysse, Germain, Hall, & Monk, 2015), and thus, the value in assessing the efficacy of this intervention on subjective sleep parameters stands strongly. Further, actigraphy, while validated for estimating TST and efficiency, may overestimate sleep duration in individuals with insomnia due to misclassification of low-movement periods as sleep (Walia & Mehra, 2019). Conversely, sleep diaries depend on subjective reporting, which, as mentioned above, is influenced by participants’ perceptions, cognitive biases, and potential recall errors (Martin & Hakim, 2011). These inherent differences between the two measurement methods can contribute to the observed discrepancies. Despite these limitations, focusing on sleep perception is clinically relevant because subjective experiences often drive treatment-seeking behaviour and influence therapeutic outcomes. Indeed, a study by Janků, Šmotek, Fárková, and Kopřivová (2020) demonstrated that two groups with differing sleep perceptions responded similarly to CBT-I. This finding underscores the importance of considering both subjective and objective measures when evaluating treatment efficacy, because they provide complementary insights into sleep patterns and perceptions. Future research should explore this discrepancy further, bearing in mind that this dCBT-I + ER may be – like many other CBT-I programmes, more successful in improving people’s perception of sleep than sleep parameters as measured by actigraphy.

Findings in this study also highlight an interesting, yet expected result patterns, as TST changes were not significantly different between the two groups after the intervention. While CBT-I significantly improved SE by reducing the time in bed, SOL, and WASO, the total sleep duration remained largely unchanged, averaging 6 hours and 30 minutes post-intervention. This outcome aligns with prior research, which shows that CBT-I primarily enhances sleep quality and efficiency rather than substantially increasing TST. For instance, prior reviews have demonstrated that despite significant enhancements in SE, only modest increases in total sleep duration, typically around 20 minutes or less, are often observable in the short-term particularly (Trauer, Qian, Doyle, Rajaratnam, & Cunnington, 2015). These results underscore the notion that CBT-I’s active ingredients may lie in its ability to consolidate sleep and restructure maladaptive sleep behaviours rather than directly extend sleep duration. The limited change in sleep duration also reflects the intentional focus of sleep restriction therapy within CBT-I, which optimises sleep pressure and efficiency but inherently caps sleep time during the treatment phase, with potential extensions to sleep duration more likely to be visible beyond the 8-week study period. Future research could explore modifications to CBT-I, such as progressive extensions of time in bed post-treatment as part of a longer follow-up period, to evaluate whether this approach could enhance total sleep duration while maintaining improvements in SE. Finally, to avoid survey fatigue on participants, the number of questions had to be limited to absolute essentials. However, future trials should collect information on parenthood and the number of children and their age, and control for factors like having young children in the home and how that would impact participants’ sleep or progress on the intervention.

## Conclusions

This trial demonstrated that digital hybrid CBT-I + ER effectively reduced insomnia, depression, and anxiety symptoms in working adults. The intervention, delivered through workplaces by non-clinical staff, suggests preliminary validity and efficacy for dCBT-I + ER as a solution beyond routine clinical care. Further investigation with longer-term follow-ups is needed to enhance confidence in its long-term efficacy and cost-effectiveness.

## Supporting information

Moukhtarian et al. supplementary material Moukhtarian et al. supplementary material

## Data Availability

The data underlying this study are available upon request. Please contact the corresponding for access to the data.

## Footnotes

1 In the absence of an EQ-5D-5L value set for England that is approved for use by the National Institute for Health and Care Excellence, our report focuses on mapping 5L responses to the 3L value set to compute a summary index score.

2 In this article, we do not report on uncontrolled follow-up data of the control group when they are enrolled in the treatment after the 8 weeks wait-time.

## Supplementary material

The supplementary material for this article can be found at http://doi.org/10.1017/S0033291725000194.
