# Supplementary material for: Digital CBT for insomnia and emotion regulation in the workplace: a randomised waitlist-controlled trial
Source: Psychol Med. 2025 Feb 17;55:e52. doi: 10.1017/S0033291725000194 (PMC12080640; doi:10.1017/S0033291725000194)
Supplement: Moukhtarian et al. supplementary material [file S0033291725000194sup001.docx]

## Appendix 1: Assessment of safety

The likelihood of adverse events (AE) occurring during this trial was low. Previous studies have shown that daytime sleepiness, tiredness and vigilance impairment may increase during sleep restriction therapy (SRT), which is one of the behavioural components of CBT-I (Kyle et al., 2014). For the full procedure on how AE and Serious AE (SAE) were reported, see trial protocol (Moukhtarian et al., 2022). AE and SAE during the study period that may or may not have been due to the intervention were recorded by the research team to check for any patterns or trends in events.

The table below summarises the adverse event reports received during the trial. None were judged serious or related to the treatment.

Table 1 Reports of Adverse Events

| **Adverse Event Description** | **Related/Unrelated** | **Outcome*** |
| --- | --- | --- |
| Employee signed off work with mental health burnout | Unrelated | Withdrew from the trial |
| Head injury sustained during the trial | Unrelated | Continued in the trial |
| Signed off work, suspected vitamin deficiency | Unrelated | Continued in the trial |
| Signed off work due to stress after first therapy appointment | Unrelated | Withdrew from the trial |
| Rash from wearing the sleep tracker | Unrelated | Continued in the trial |

*None of the participants withdrew consent, and their data was therefore not deleted.

## Appendix 2: Variable distributions

Normality of all variables in the study were examined both graphically through histograms and Q-Q plots, and analytically reporting on the Shapiro-Wilk test.

**Primary outcomes**

GAD-7

W (159) = .96, *p* < .01


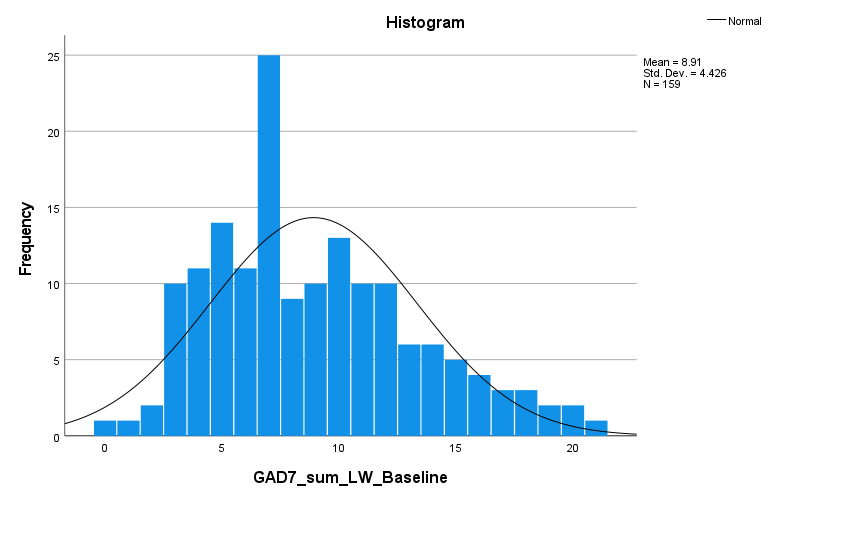


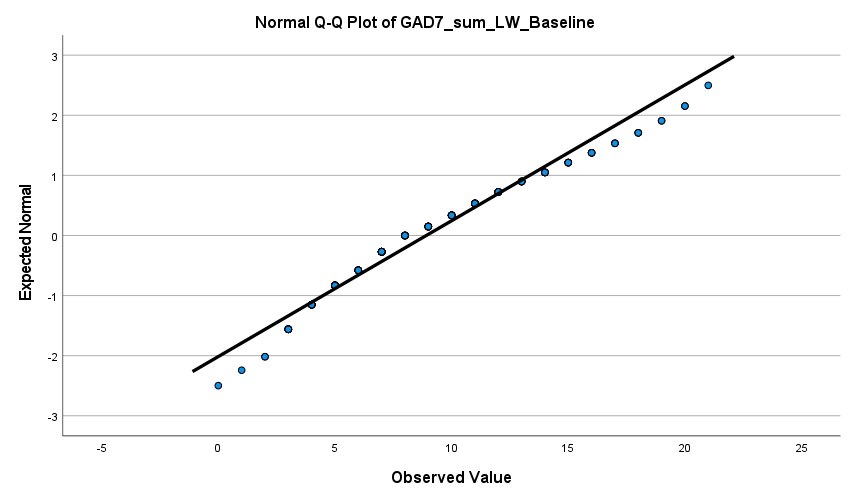


PHQ-9

W (159) = .97, *p* < .001


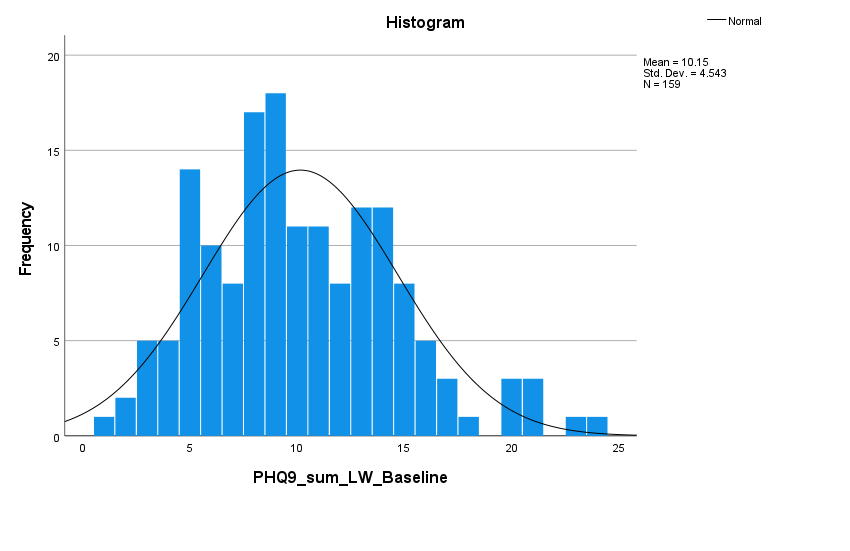


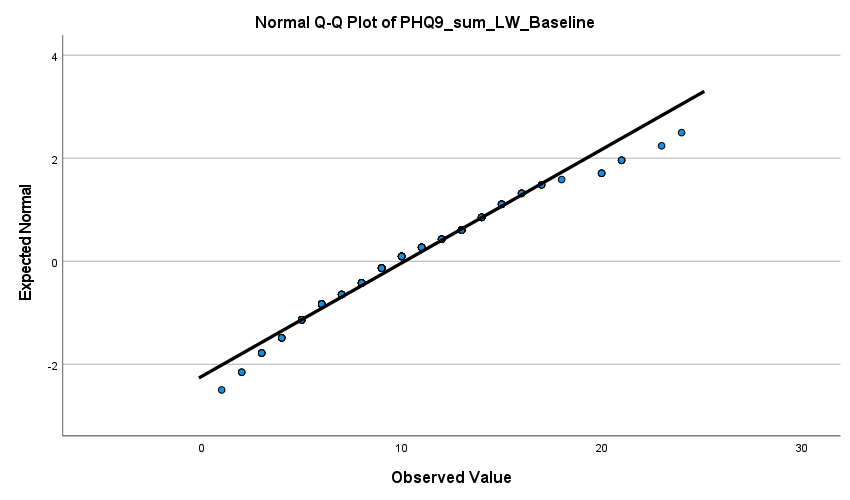


ISI

W (159) = .99, *p* = .29
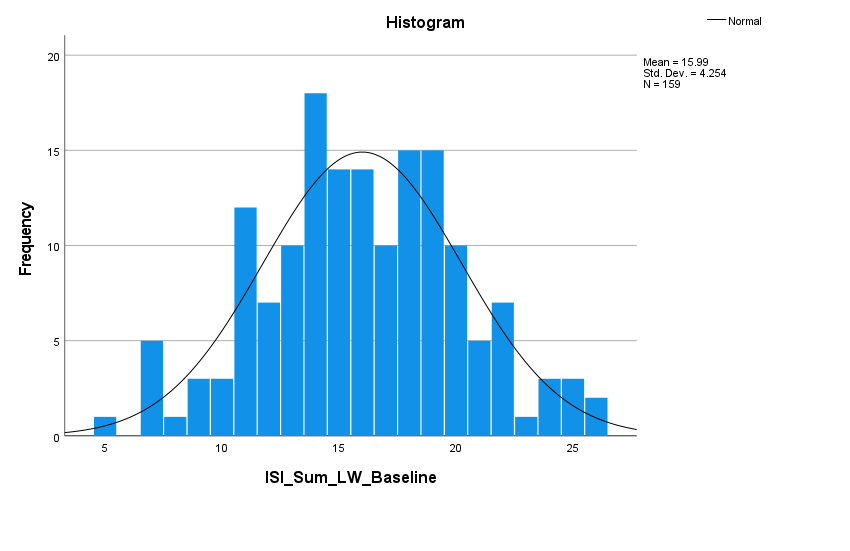


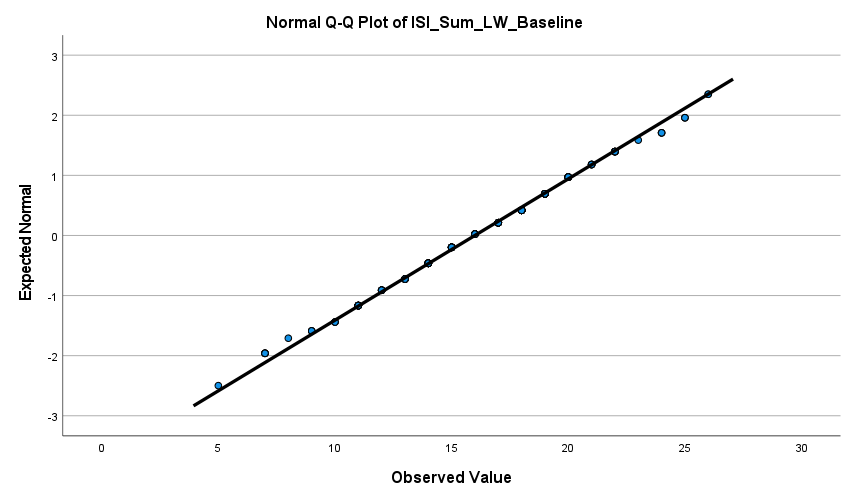


**Secondary questionnaire outcomes**

IJSS

W (159) = .99, *p* = .12


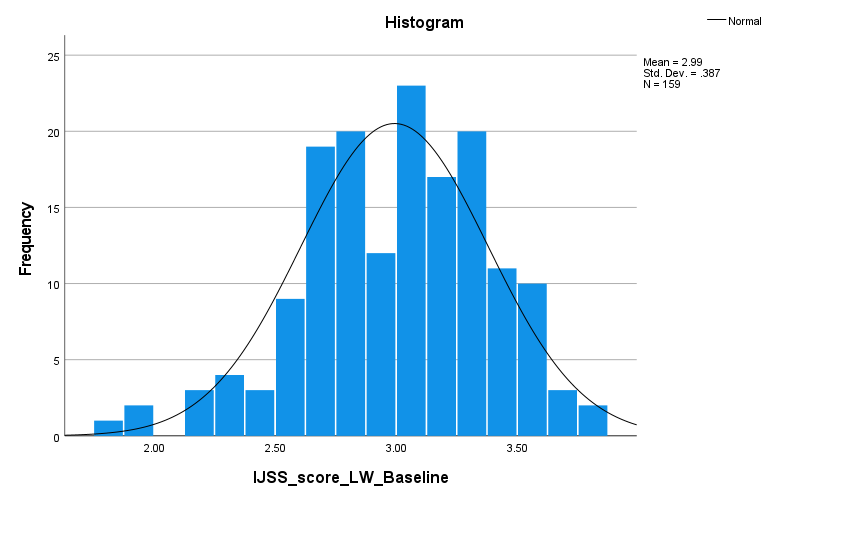


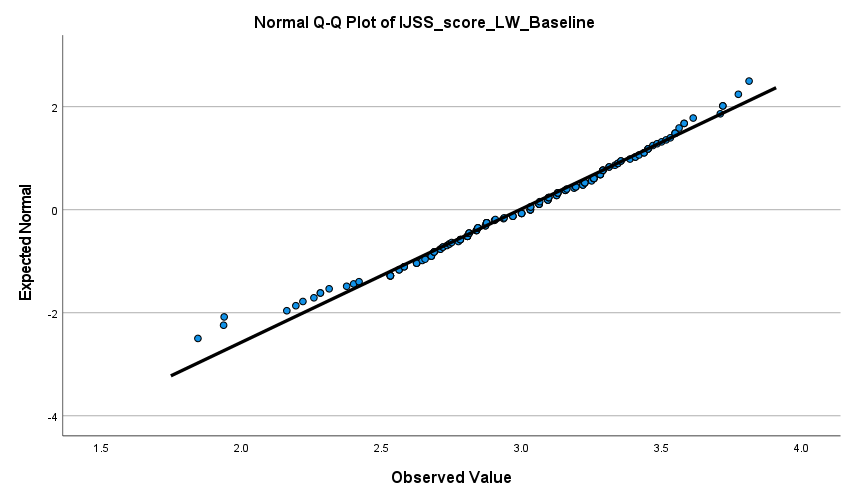


WEMWBS

W (156) = .98, *p* = .05


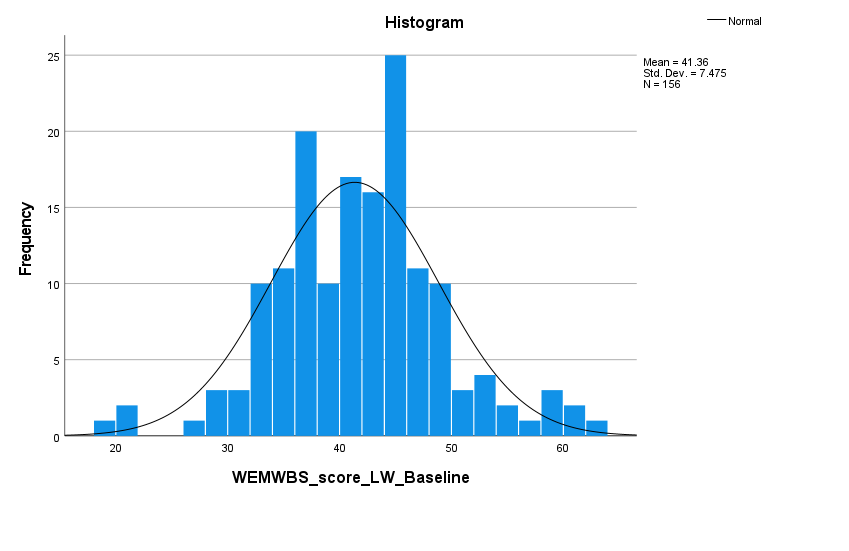


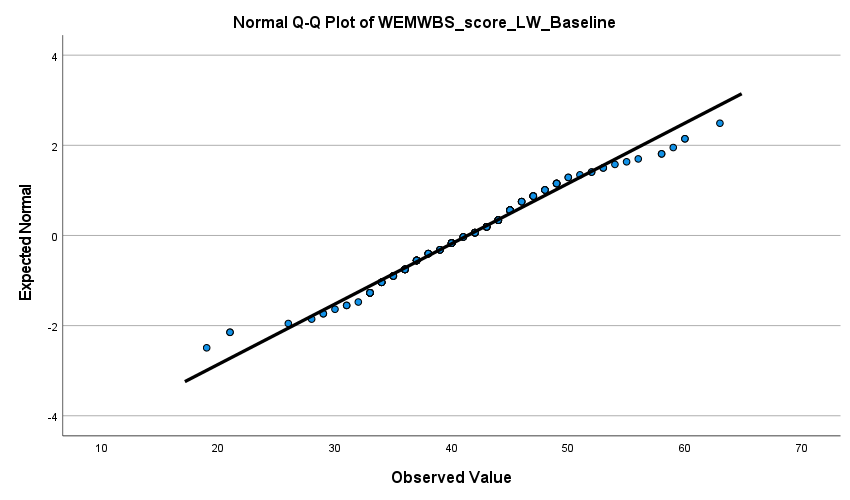


WPAI1-WTM

W (158) = .29, *p* < .001


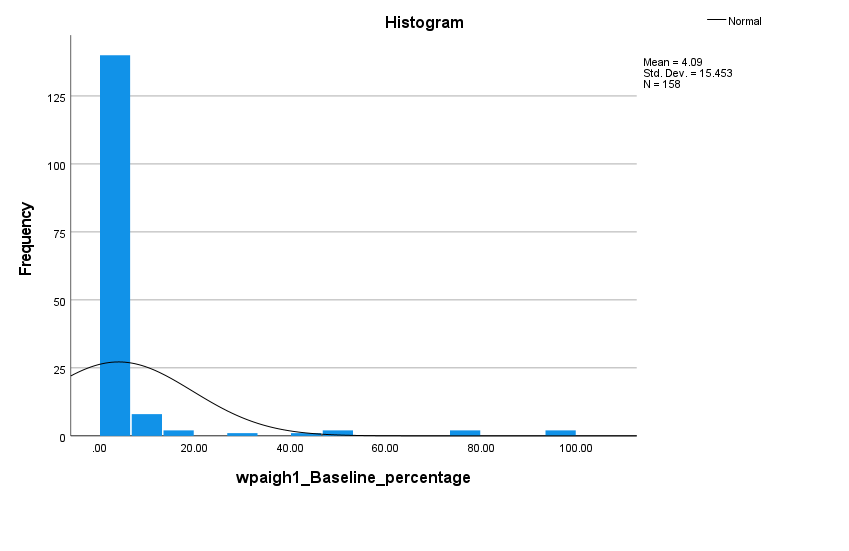


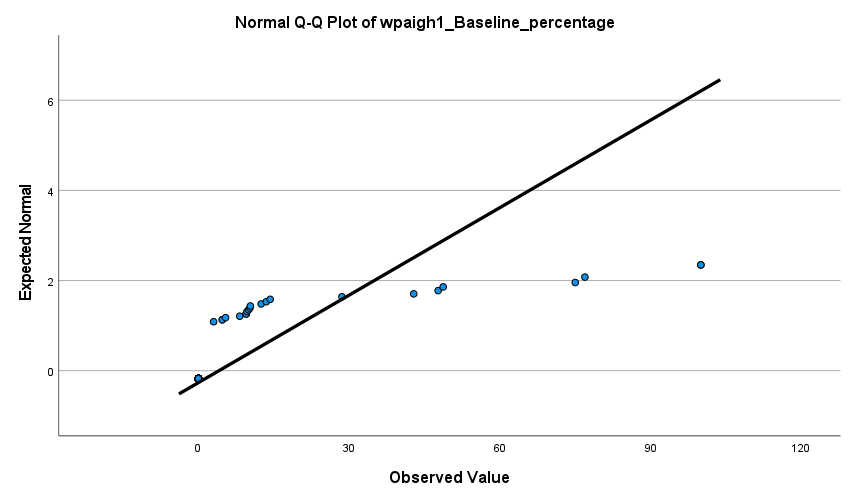


WPAI2-IWW

W (159) = .93, *p* < .001


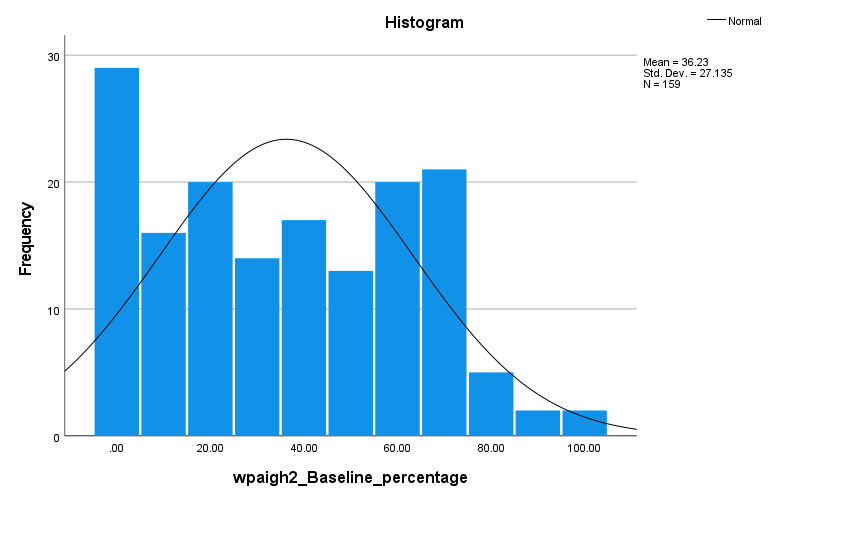


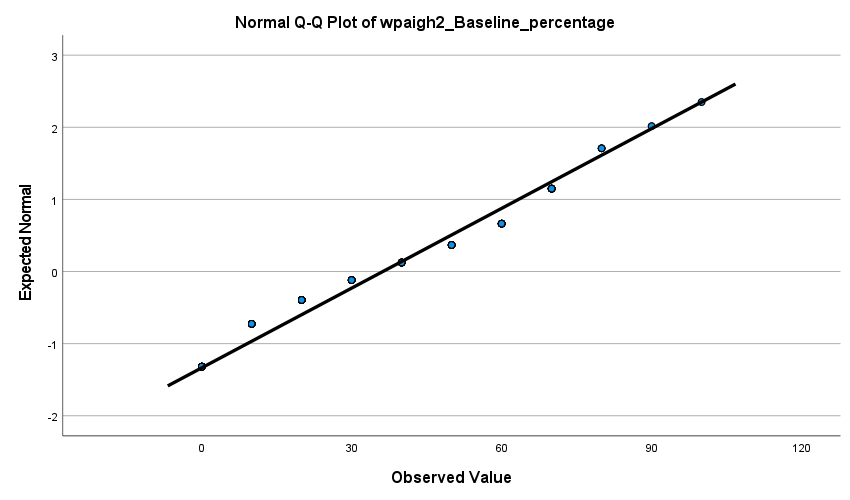


WPAI3-OWI

W (158) = .93, *p* < .001


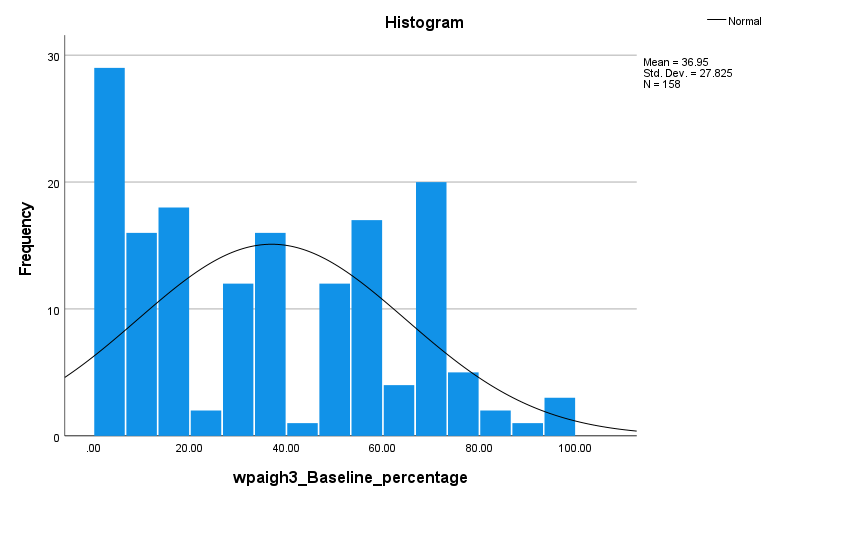


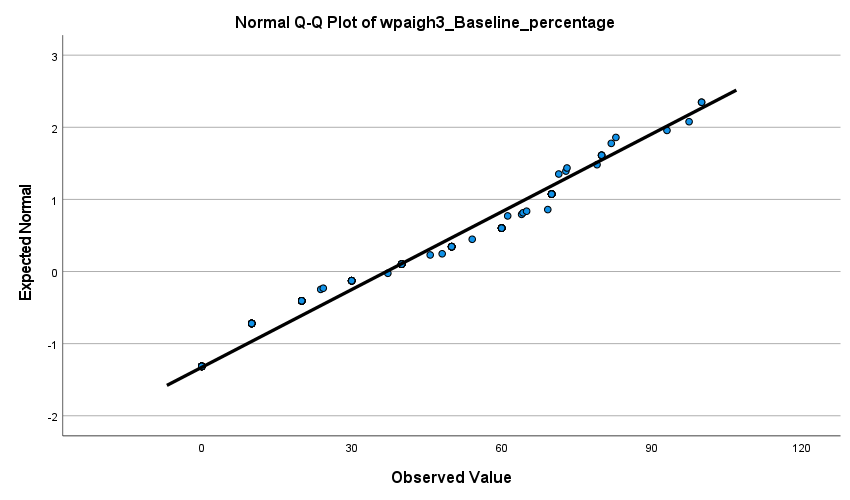


WPAI4-AI

W (159) = .93, *p* < .001


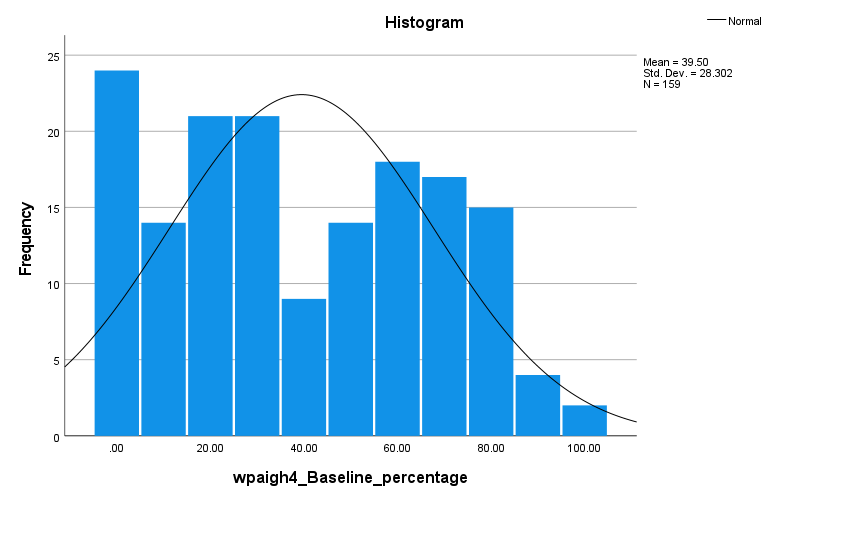


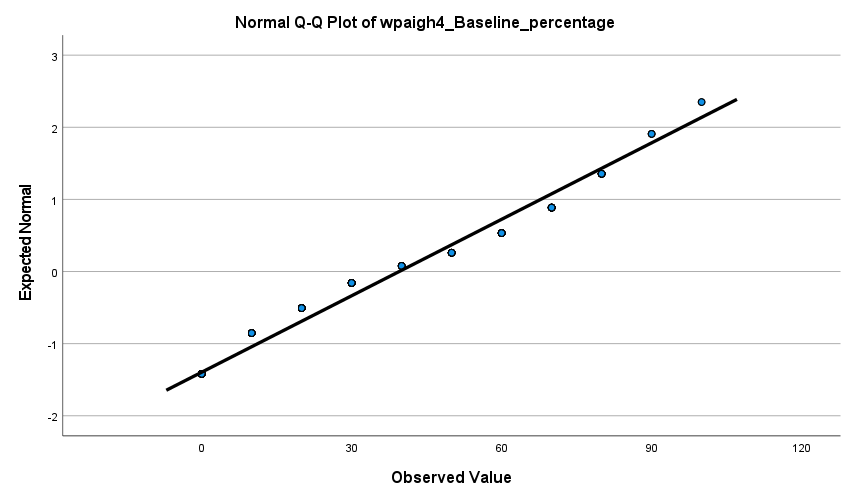


EQ5D – Utility score

W (155) = .92, *p* < .001

**
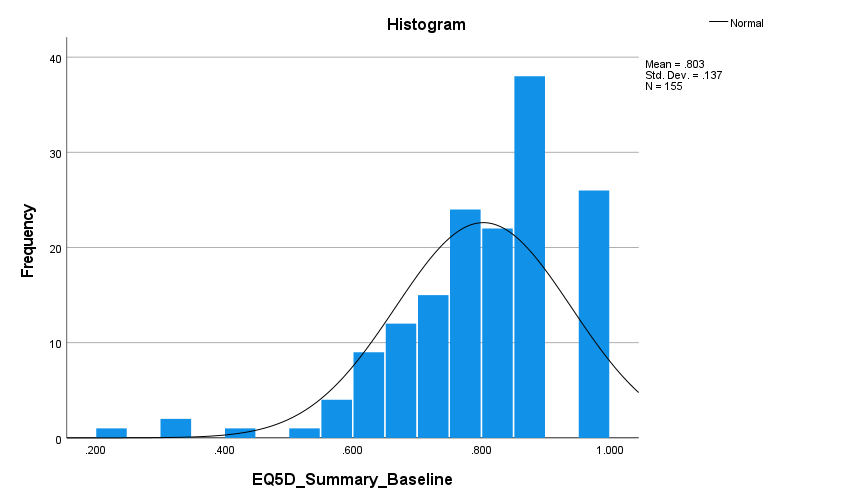
**

**
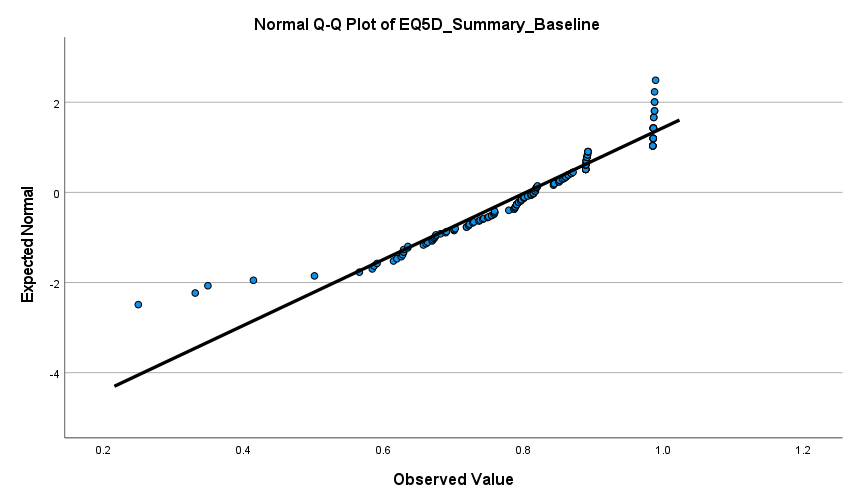
**

EQ5D – VAS score

W (156) = .94, *p* < .001


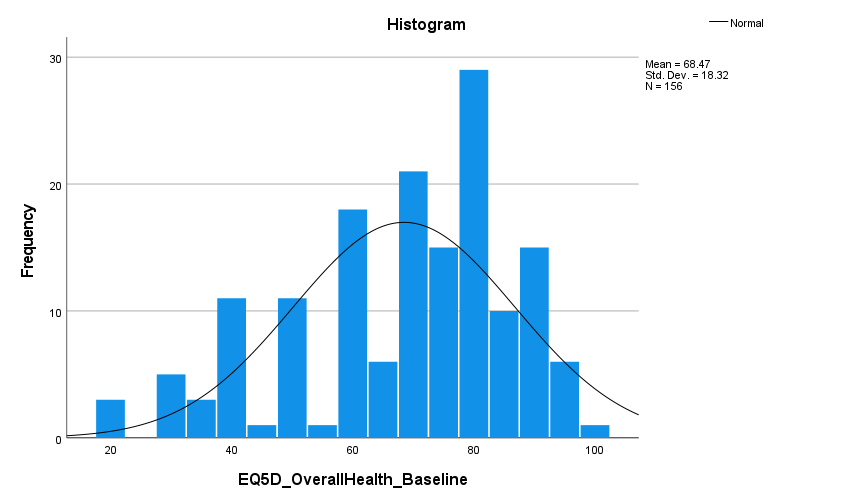


**
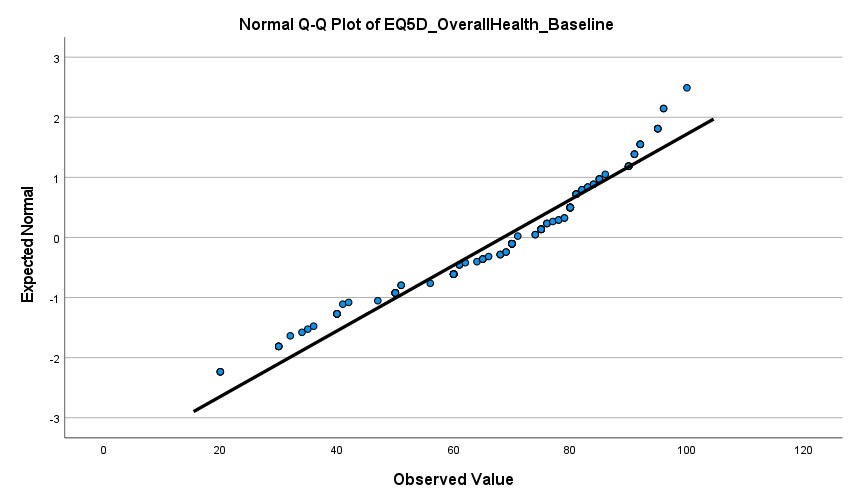
**

**Secondary outcomes- objective sleep tracker**

Sleep efficiency

W (119) = .84, *p* < .001


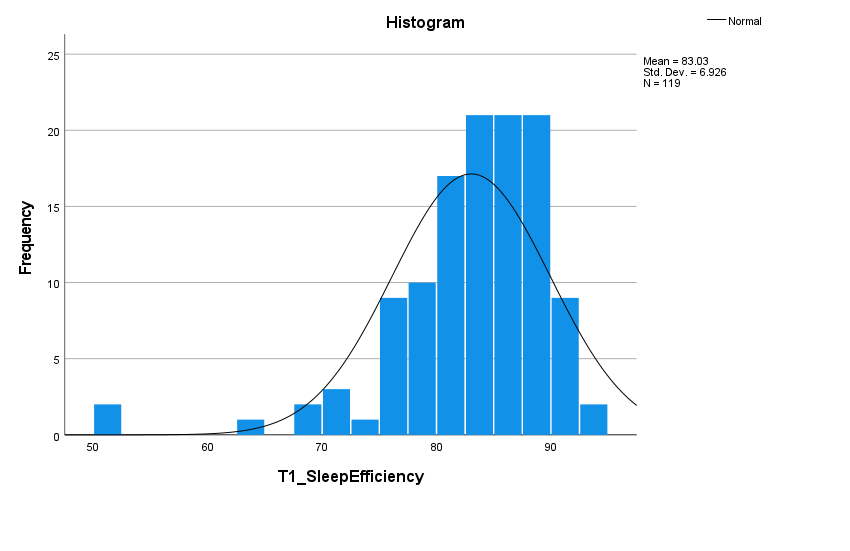


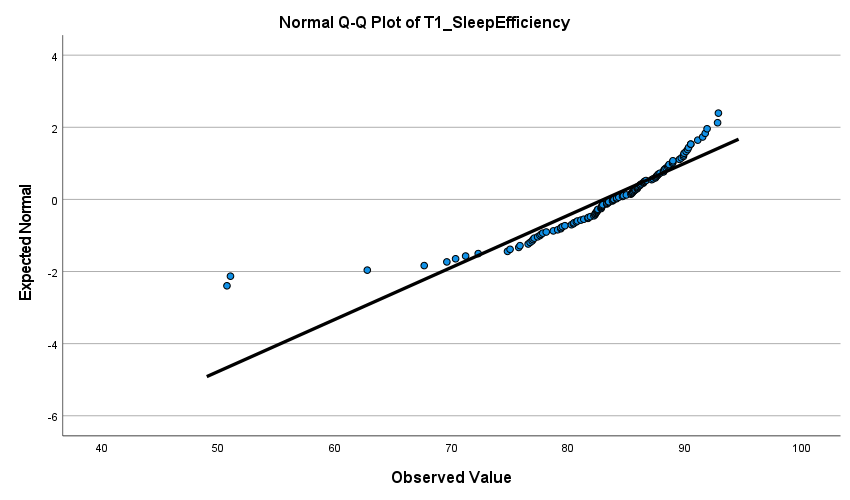


Total time in bed (TIB) in minutes

W (119) = .95, *p* < .001


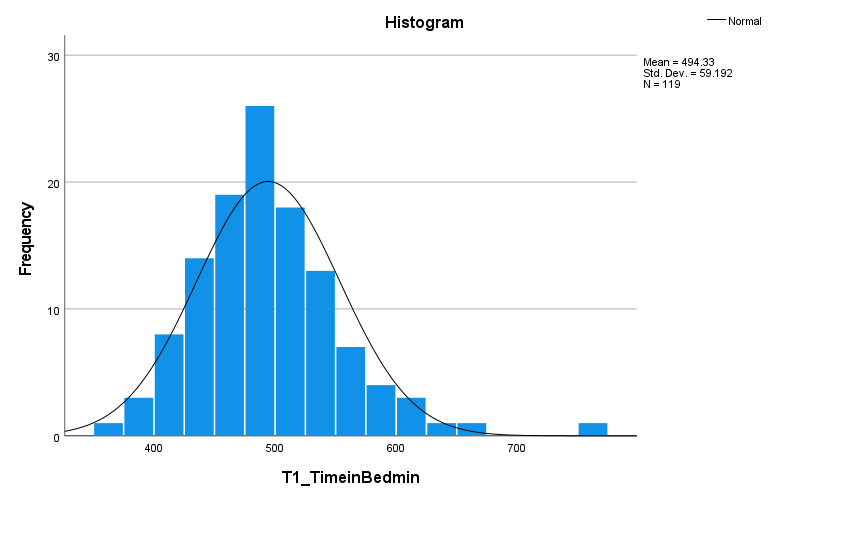


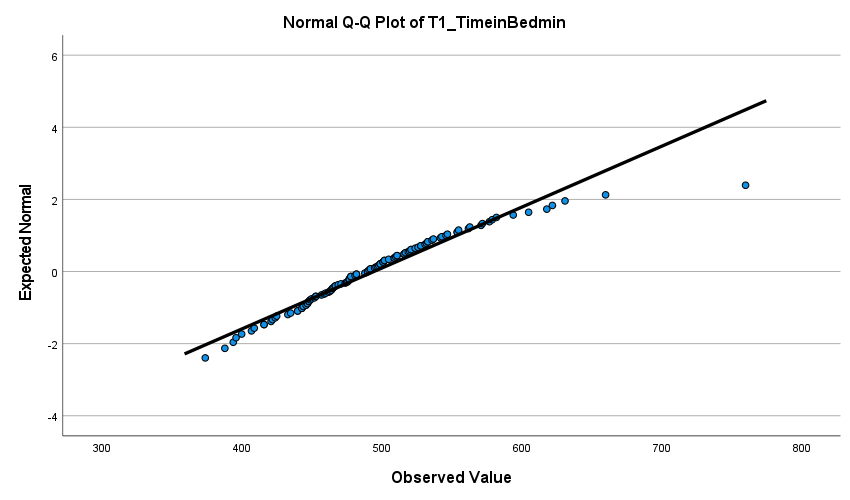


Total sleep time (TST) in minutes

W (119) = .98, *p* = .10


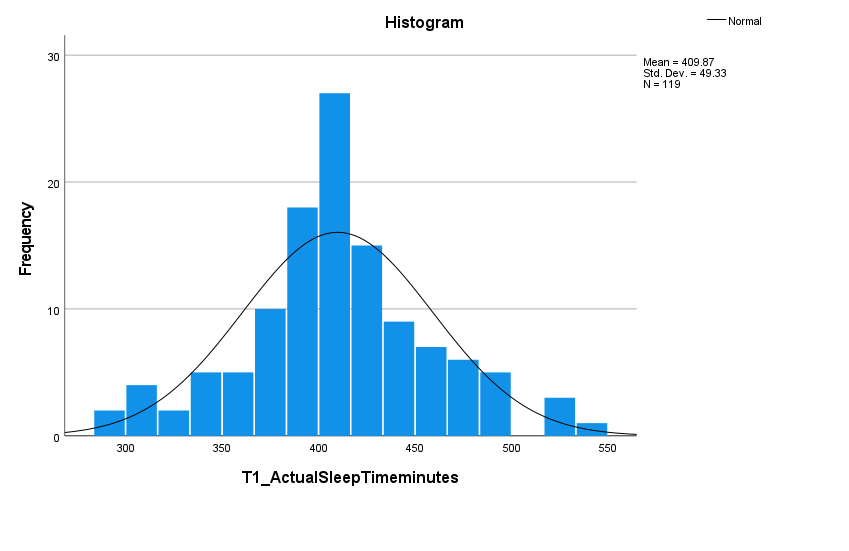


**Normal Q-Q Plot of T1_TotalSleepTimeminutes**

**T1_TotalSleepTimeminutes**


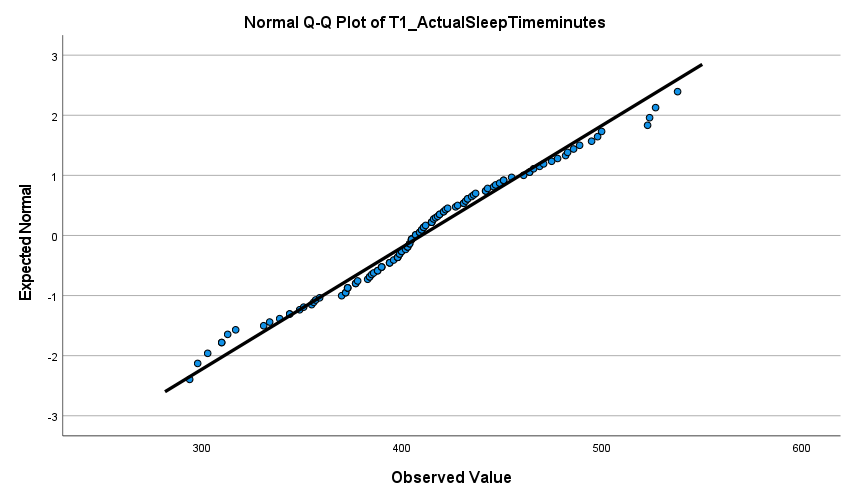


Total wake time in minutes

W (119) = .77, *p* < .001


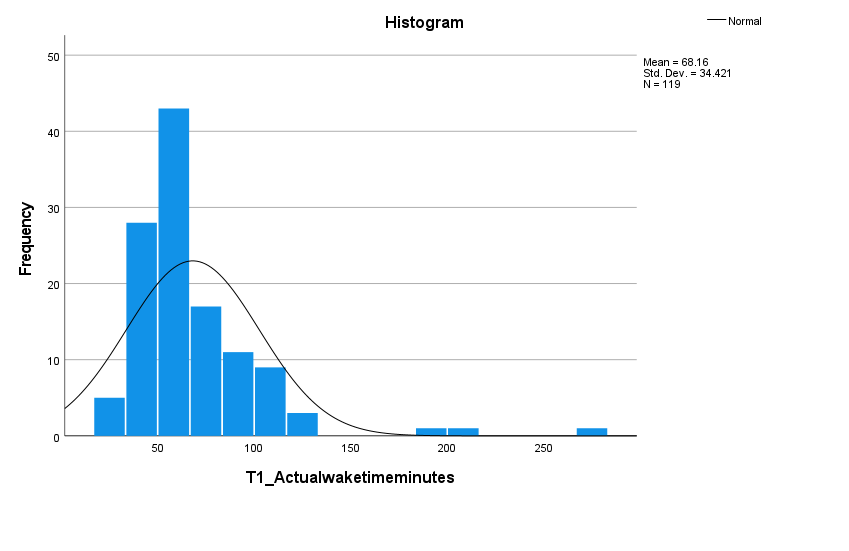


**Normal Q-Q Plot of T1_TotalWakeTimeminutes**

**T1_TotalWakeTimeTimeminutes**


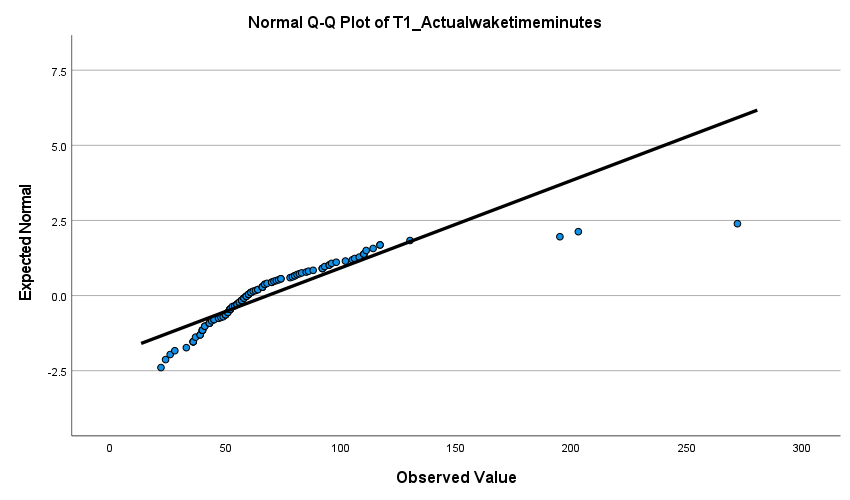


**Secondary outcomes- self-reported sleep diary**

Sleep efficiency

W (102) = .94, *p* < .001


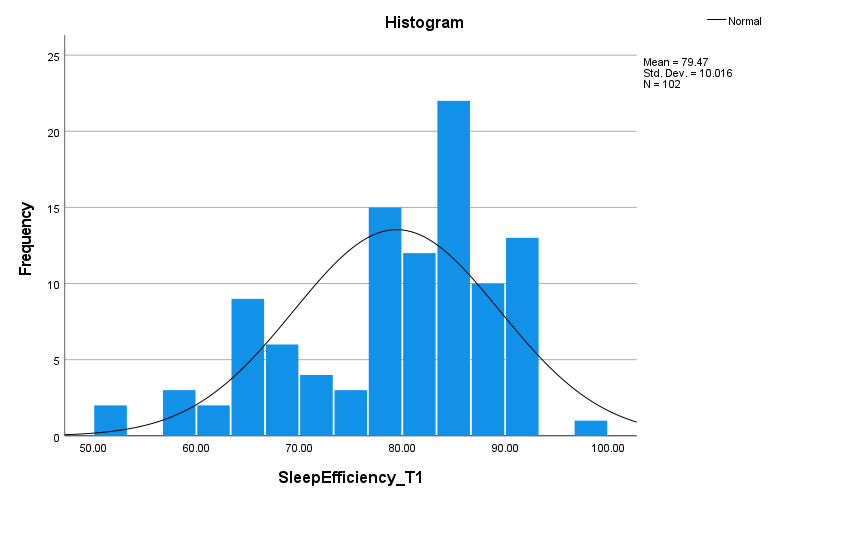


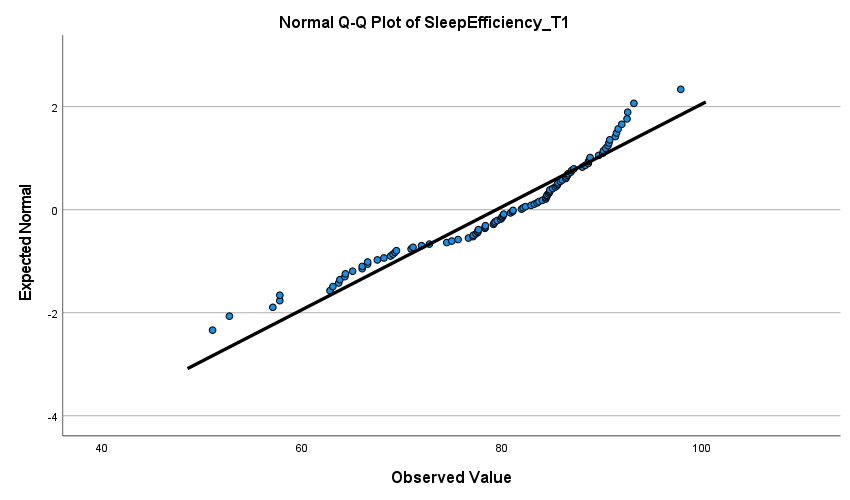


Sleep quality

W (102) = .98, *p* = .18


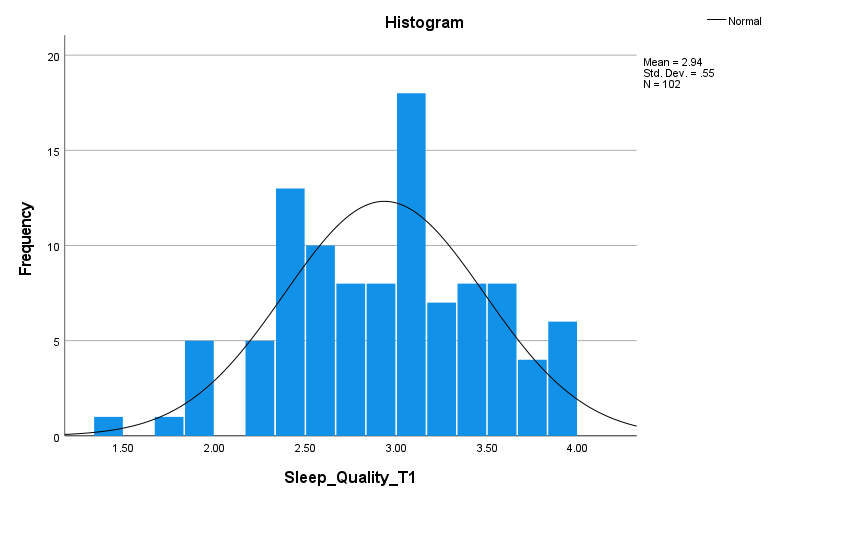


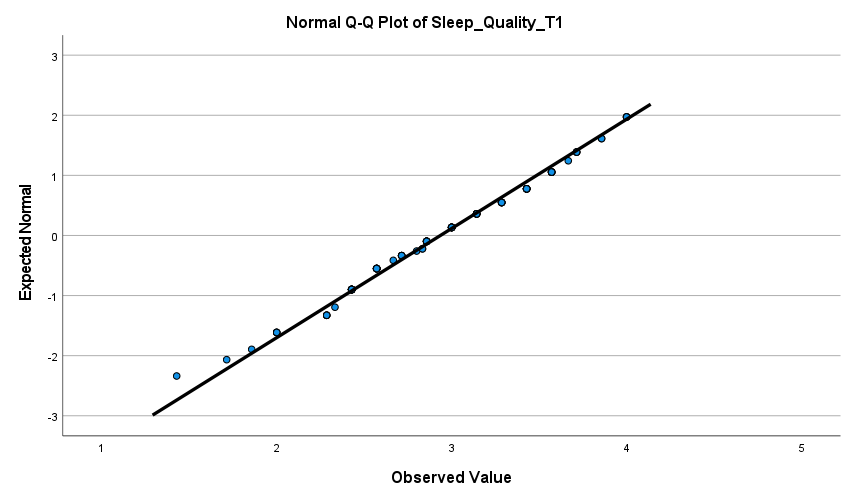


Sleep Onset Latency (SOL)

W (102) = .88, *p* < .001


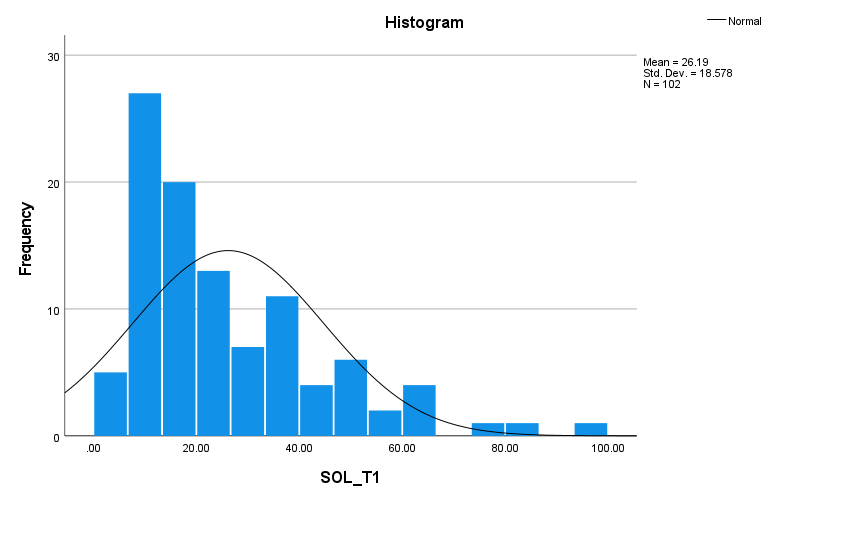


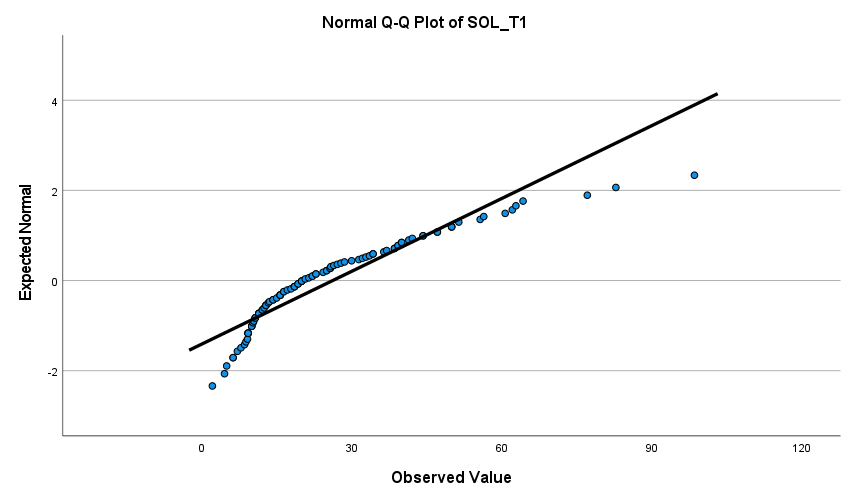


Wake After Sleep Onset (WASO)

W (102) = .87, *p* < .001


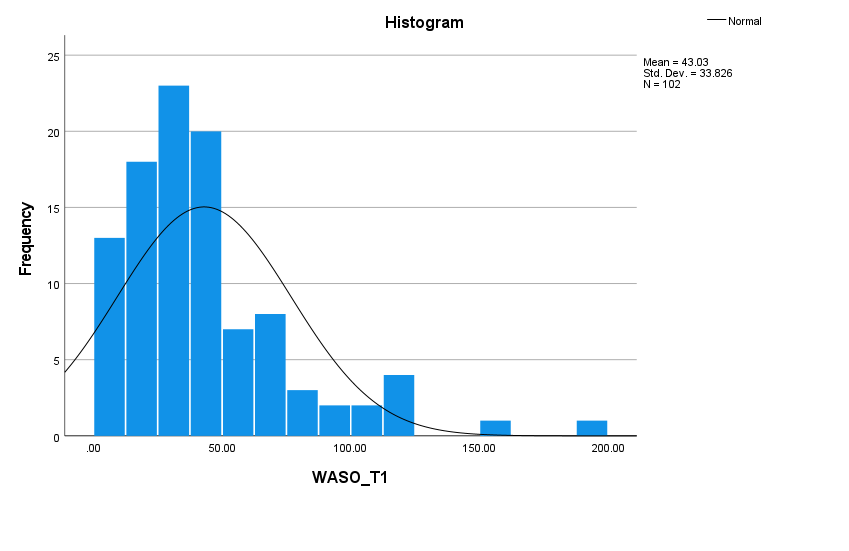


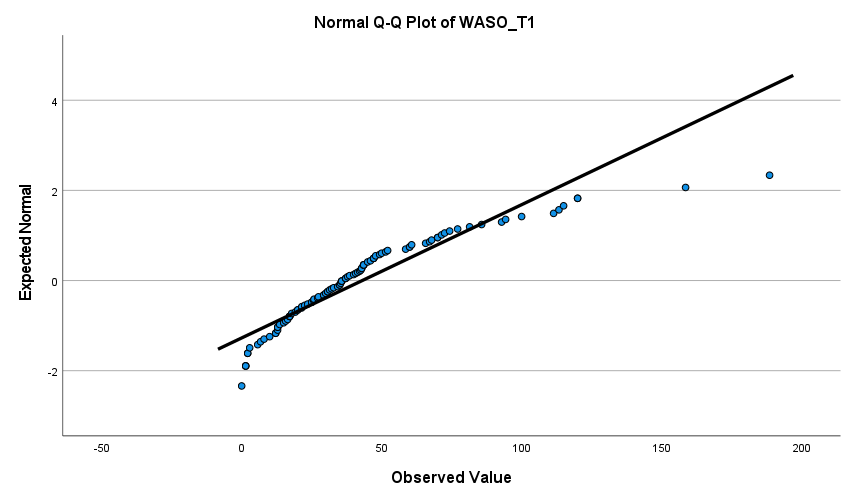


Time in bed (TIB)

W (102) = .96, *p* = .005


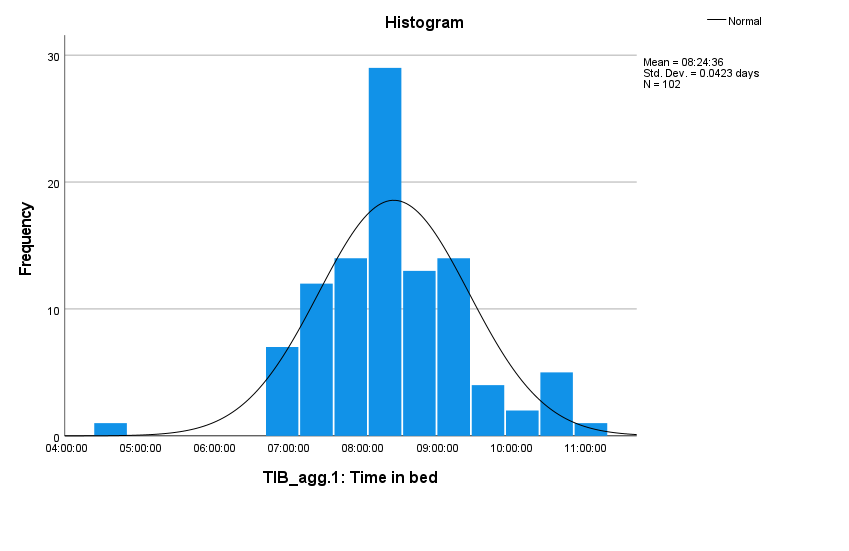


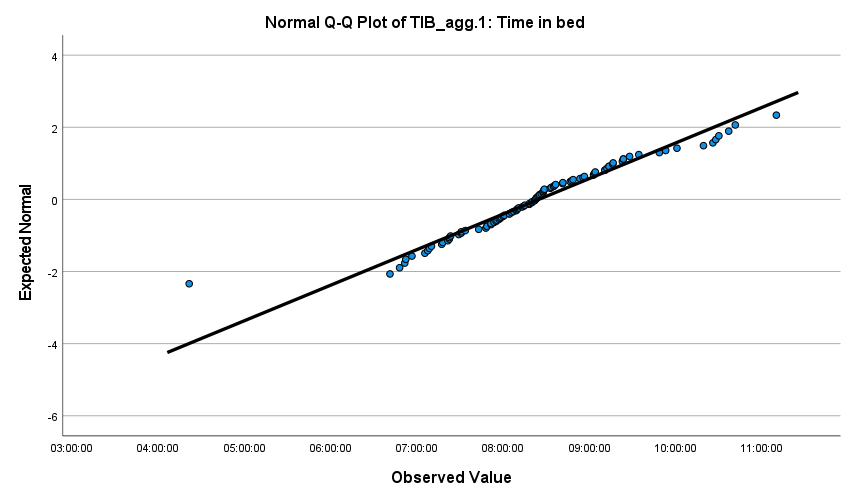


Total sleep time (TST)

W (102) = .98, *p* = .16


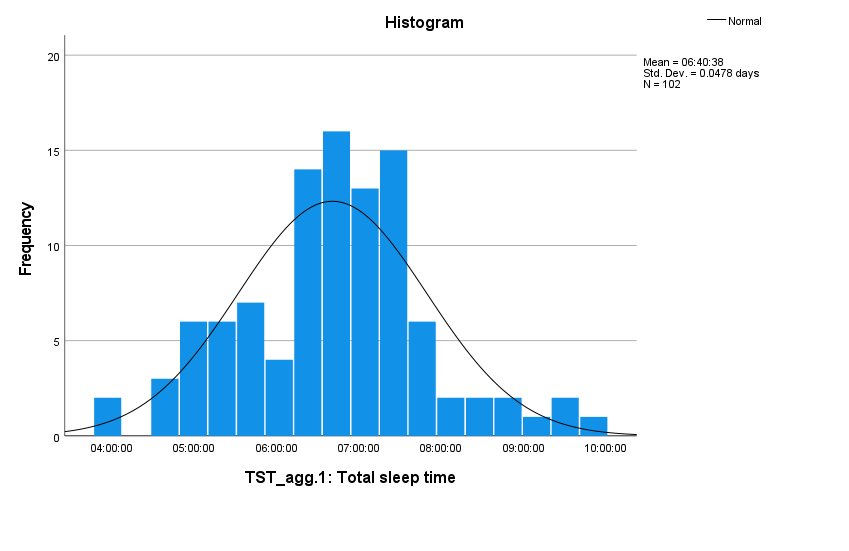


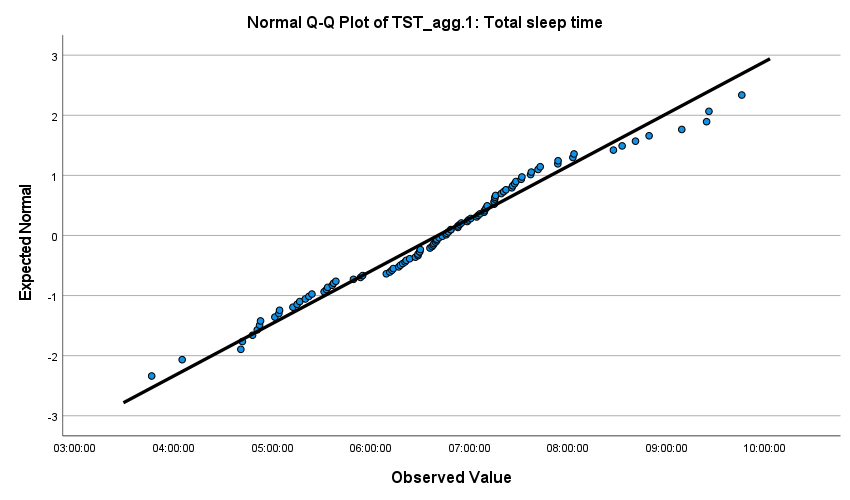


## Appendix 3: Missingness

We created a categorical variable to indicate missingness and non-missingness at T2 (1= missing at T2, 2= complete at T2). We then ran multinomial logistic regression models using missingness as the dependent variable, allocation as factor and each of the outcome measures as covariates to predict missingness with the variables at T1 (i.e. does symptom severity on ISI at baseline predict missingness/drop out at T2).

We report below descriptives divided by allocation across the subgroup of people with missing or non-missing T2 outcomes, and *p* values from the model fitting. Using the Bonferroni corrected *p* value of **0.01667, of all the variables, only the three subscales of the WPAI appear to significantly predict missingness (attrition).**

Table 2 Descriptive statistics and test statistic of the multinomial logistic regression models of all primary and secondary outcomes predicting missingness across the two groups.

|  | **Predicting missingness (*p*-value)** | **dCBTI+ER**  **(n= 79)**  **M(SD)** | | **Control**  **(n= 80)**  **M(SD)** | |
| --- | --- | --- | --- | --- | --- |
|  |  | **Missing**  **(n = 16)** | **Not missing**  **(n = 63)** | **Missing**  **(n = 7)** | **Not missing**  **(n = 73)** |
| **Demographics** |  |  |  |  |  |
| Age, y | 0.110 | 43.88 (9.85) | 44.78 (8.98) | 42.71 (11.70) | 42.53 (9.55) |
| Sex, No. (%) | 0.110 |  |  |  |  |
| Women |  | 12 (75.00) | 47 (74.60) | 5 (71.40) | 58 (79.50) |
| Men, Other |  | 4 (25.00) | 16 (25.40) | 2 (28.60) | 15 (20.50) |
| Ethnicity, No. (%) | 0.036 |  |  |  |  |
| White |  | 14 (87.50) | 50 (79.40) | 7 (100.00) | 57 (78.10) |
| Black, Asian, Mixed, Other |  | 2 (12.50) | 13 (20.60) | 0 (0.00) | 16 (21.90) |
| Relationship status, No. (%) | 0.087 |  |  |  |  |
| Married |  | 6 (37.50) | 33 (52.40) | 5 (71.40) | 38 (52.10) |
| Co-habiting |  | 2 (12.50) | 12 (19.00) | 1 (14.30) | 15 (20.50) |
| Single |  | 4 (25.00) | 10 (15.90) | 1 (14.30) | 13 (17.80) |
| Divorced, Separated, Widowed, Other |  | 4 (25.00) | 8 (12.70) | 0 (0.00) | 7 (9.60) |
| Hours of work | 0.113 | 37.56 (5.34) | 37.59 (7.02) | 37.71 (3.86) | 37.27 (6.89) |
| Education level, No. (%) | 0.091 |  |  |  |  |
| Higher education |  | 11 (68.80) | 38 (60.30) | 1 (14.30) | 39 (53.40) |
| Lower education |  | 5 (31.30) | 25 (39.70) | 6 (85.70) | 34 (46.60) |
| Income No. (%) | 0.066 |  |  |  |  |
| £10,000-£29,999 |  | 5 (31.30) | 7 (11.10) | 1 (14.30) | 19 (26.00) |
| £30,000 or higher |  | 11 (68.80) | 56 (88.90) | 6 (85.70) | 54 (74.00) |
| **Primary outcomes** |  |  |  |  |  |
| Insomnia (ISI) | 0.078 | 16.19 (3.73) | 16.38 (4.25) | 18.29 (3.09) | 15.40 (4.41) |
| Depression (PHQ-9) | 0.11 | 9.50 (4.94) | 10.02 (4.38) | 10.71 (4.54) | 10.36 (4.67) |
| Anxiety (GAD-7) | 0.077 | 8.13 (4.29) | 8.84 (4.64) | 8.00 (3.96) | 9.23 (4.35) |
| **Categorical** |  |  |  |  |  |
| Insomnia | 0.082 |  |  |  |  |
| No clinically significant insomnia |  | 0 (0.00) | 2 (3.20) | 0 (0.00) | 4 (5.50) |
| Subthreshold insomnia |  | 6 (37.50) | 17 (27.00) | 1 (14.30) | 30 (41.10) |
| Clinical insomnia |  | 10 (62.50) | 44 (69.80) | 6 (85.70) | 39 (53.40) |
| Depression (PHQ-9) | 0.111 |  |  |  |  |
| Normal |  | 3 (18.80) | 6 (9.50) | 0 (0.00) | 4 (5.50) |
| Mild |  | 5 (31.30) | 26 (41.30) | 4 (57.10) | 32 (43.80) |
| Mild-moderate |  | 5 (31.30) | 21 (33.30) | 2 (28.60) | 26 (35.60) |
| Moderate |  | 3 (18.80) | 7 (11.10) | 0 (0.00) | 7 (9.60) |
| Severe |  | 0 (0.00) | 3 (4.80) | 1 (14.30) | 4 (5.50) |
| Anxiety (GAD-7) | 0.083 |  |  |  |  |
| No to low risk |  | 5 (31.30) | 10 (15.90) | 1 (14.30) | 9 (12.30) |
| Mild |  | 5 (31.30) | 30 (47.60) | 3 (42.90) | 31 (42.50) |
| Moderate |  | 4 (25.00) | 14 (22.20) | 3 (42.90) | 24 (32.90) |
| Severe |  | 2 (12.50) | 9 (14.30) | 0 (0.00) | 9 (12.30) |
| **Secondary outcomes** |  |  |  |  |  |
| Work productivity (WPAI:GH) |  |  |  |  |  |
| WPAIGH1 | **< 0.001** | 5.39 (18.68) | 1.23 (4.63)^3^ | 34.69 (47.28) | 3.29 (12.02) |
| WPAIGH2 | **0.013** | 39.38 (30.87) | 32.86 (25.87) | 61.43 (36.71) | 36.03 (25.59) |
| WPAIGH3 | **0.010** | 40.18 (31.89) | 32.54 (25.47)^3^ | 63.27 (37.52) | 37.48 (26.89) |
| WPAIGH4 | 0.094 | 35.00 (30.98) | 37.30 (26.35) | 57.14 (28.70) | 40.68 (29.17) |
| Job satisfaction (IJSS) | 0.111 | 2.94 (0.39) | 3.02 (0.40) | 3.08 (0.26) | 2.97 (0.39) |
| Well-being (WEMWBS) | 0.098 | 42.80 (6.60)^2^ | 41.71 (7.48) | 43.86 (9.35) | 40.49 (7.48)^1^ |
| Quality of life (EQ-5D-5L) |  |  |  |  |  |
| EQ-5D-5L utility score | 0.084 | 0.85 (0.16) | 0.81 (0.10)^4^ | 0.72 (0.20) | 0.80 (0.15) |
| EQ-5D-5L VAS | 0.091 | 73.38 (21.74) | 68.98 (16.70)^5^ | 59.29 (19.51) | 67.86 (18.70) |

Footnote:

1. n = 71
2. n = 15
3. n = 62
4. n = 59
5. n = 60

## Appendix 4: Caseness

We defined caseness on the outcomes according to the Improving Access to Psychological Therapies (IAPT) framework. This is defined as those with a PHQ-9 score of at least 10, a GAD-7 score of at least 8 (NHS England, 2018), and an ISI score of at least 8 (Harvey et al., 2014). Binary (yes/no) variables were computed for baseline and week 8 for these three outcomes indicating caseness. Clinically significant change (CSC), or symptom reduction of reliable change was also explored; this is defined as those with a reduction of at least 8 points on the ISI (Morin et al., 2011), 4 points on the GAD and 6 points on the PHQ-9 (England, 2018). We created change score variables from baseline to week 8 for the primary outcome measures, then created binary (yes/no) CSC variables with the above benchmark scores. Differences of CSC between the control and treatment groups are tested by Chi-Square tests.

## Appendix 5: Fixed effects of group x time interactions on secondary outcomes

- WPAI-WTM: F(1, 133.48) = 0.66, *p =* 0.4184
- WPAI-IWW: F(1, 134) = 1.99, *p =* 0.1610
- WPAI-OWI: F(1, 131.92) = 1.09, *p =* 0.2985
- WPAI-AI: F(1, 134) = 4.82, ***p =* 0.0298** (did not withstand Bonferroni correction)
- IJSS: F(1, 134) = 0.16, *p* = 0.6897
- EQ-5D-5L Utility: F(1, 130.24) = 1.83, *p =* 0.17818
- E5-5D-5L VAS: F(1, 131.34) = 3.35, *p =* 0.06939
- Time in Bed: F(1, 101) = 13.34, *p =* **0.0004**
- Sleep Onset Latency: F(1, 101) = 11.21, ***p =* 0.0011**
- Wake After Sleep Onset: F(1, 101) = 6.78, *p =* **0.0106**
- Sleep efficiency: F(1, 101) = 12.52, ***p =* 0.0006**
- Sleep quality: F(1, 100.19) = 14.25, ***p =* 0.0003**

References

NHS England, (2018). *The Improving Access to Psychological Therapies Manual*. <https://www.england.nhs.uk/publication/the-improving-access-to-psychological-therapies-manual/>

Harvey, A. G., Bélanger, L., Talbot, L., Eidelman, P., Beaulieu-Bonneau, S., Fortier-Brochu, É., Ivers, H., Lamy, M., Hein, K., Soehner, A. M., Mérette, C., & Morin, C. M. (2014). Comparative efficacy of behavior therapy, cognitive therapy, and cognitive behavior therapy for chronic insomnia: a randomized controlled trial. *J Consult Clin Psychol*, *82*(4), 670-683. <https://doi.org/10.1037/a0036606>

Kyle, S. D., Miller, C. B., Rogers, Z., Siriwardena, A. N., MacMahon, K. M., & Espie, C. A. (2014). Sleep Restriction Therapy for Insomnia is Associated with Reduced Objective Total Sleep Time, Increased Daytime Somnolence, and Objectively Impaired Vigilance: Implications for the Clinical Management of Insomnia Disorder. *Sleep*, *37*(2), 229-237. <https://doi.org/10.5665/sleep.3386>

Morin, C. M., Belleville, G., Bélanger, L., & Ivers, H. (2011). The Insomnia Severity Index: Psychometric Indicators to Detect Insomnia Cases and Evaluate Treatment Response. *Sleep*, *34*(5), 601-608. <https://doi.org/10.1093/sleep/34.5.601>

Moukhtarian, T. R., Patel, K., Toro, C., Russel, S., Daly, G., Walasek, L., Tang, N. K. Y., & Meyer, C. (2022). Effects of a hybrid digital cognitive-behavioural therapy for insomnia and emotion regulation in the workplace (SLEEP): study protocol for a randomised waitlist control trial. *BMJ Open*, *12*(7), e058062. <https://doi.org/10.1136/bmjopen-2021-058062>
